# Supplemental structured surveys and pre-existing detection models improve fine-scale density and population estimation with opportunistic community science data

**DOI:** 10.1038/s41598-024-61582-6

**Published:** 2024-05-14

**Authors:** Tyler A. Hallman, W. Douglas Robinson

**Affiliations:** 1 Oak Creek Lab of Biology, Department of Fisheries, Wildlife, and Conservation Sciences, Oregon State University, Corvallis, OR USA; 2 Swiss Ornithological Institute, Seerose 1, 6204 Sempach, Switzerland; 3 Department of Biology and Chemistry, Queens University of Charlotte, Charlotte, NC USA; 4 School of Environmental and Natural Sciences, Bangor University, Bangor, LL57 2DG UK

**Keywords:** Biogeography, Conservation biology

## Abstract

Density and population estimates aid in conservation and stakeholder communication. While free and broadly available community science data can effectively inform species distribution models, they often lack the information necessary to estimate imperfect detection and area sampled, thus limiting their use in fine-scale density modeling. We used structured distance-sampling surveys to model detection probability and calculate survey-specific detection offsets in community science models. We estimated density and population for 16 songbird species under three frameworks: (1) a fixed framework that assumes perfect detection within a specified survey radius, (2) an independent framework that calculates offsets from an independent source, and (3) a calibration framework that calculates offsets from supplemental surveys. Within the calibration framework, we examined the effects of calibration dataset size and data pooling. Estimates of density and population size were consistently biased low in the fixed framework. The independent and calibration frameworks produced reliable estimates for some species, but biased estimates for others, indicating discrepancies in detection probability between structured and community science surveys. The calibration framework produced reliable population estimates with as few as 10 calibration surveys with positive detections. Data pooling dramatically decreased bias. This study provides conservationists and managers with a cost-effective method of estimating density and population.

## Introduction

Population estimates are exceptionally valuable for conservation practitioners. They provide tangible and engaging numbers that aid in communicating with stakeholders, including policy makers and the public^1–3^. Further, these estimates allow practitioners to set population-based conservation goals, monitor the effects of management actions, and identify conservation successes. Conservation organizations would benefit from the development of cost-effective methods of estimating population size at local and regional scales^4,5^.

The growth of opportunistic community science projects (also known as citizen science and participatory science) such as eBird, Birdtrack, and Ornitho, provide immense opportunities to develop cost-effective methods of population estimation. Through these community science projects, the spatial and temporal breadth of available biodiversity data has reached unprecedented levels^6,7^. To increase participation, many community science projects such as eBird, encourage contributions from observers of all skill levels and allow a large variety of survey methods to be employed. While eBird does not control when or where surveys are conducted, it is classified as a semi-structured community science database. Semi-structured databases, unlike unstructured databases (e.g., iNaturalist), require additional information during submission, such as survey protocol, time, date, and number of observers. In contrast, more structured databases (e.g., North American Breeding Bird Survey) generally use strict survey methods, predefined survey locations, and trained observers. In terms of the overall information within either project type, the sheer quantity of semi-structured data may compensate for the higher per-datum quality in structured community science projects^6^.

Increased participation through the use of less strict protocols, however, is not without drawbacks. Persistent questions of data quality fuel ongoing research on statistical methods that make better use of semi-structured community science data. To date, extensive methods have been developed to improve the performance of community science based species distribution models^8–10^. Conservation planning based on abundance, however, is generally more effective than based on occurrence alone^11–13^. Further, for commonly used population-based conservation goals^14^, relative or observed abundance information is insufficient as density is required for population estimation. The relative difficulty of modeling density and population size has led to the frequent use of species occurrence as a proxy for density^15,16^. Although occurrence and density of a species are linked, their relationship is complex and nonlinear, making the direct substitution of one for the other problematic^11,14^. Estimating density and population from community science data, however, presents a unique set of challenges. While abundance is increasingly available in large community science databases, densities of organisms that allow for population estimation are not.

Distribution models built on opportunistic community science data can produce predictions comparable to those informed by professional surveys, but abundance information in community science data can be considerably biased and options for estimating density are limited^17,18^. Addressing these biases, while estimating density and population from observed abundance, requires additional information. Distance sampling data, for example, can address biases through the explicit estimation of individual detection probability. Perhaps more importantly, estimates of area surveyed are essential to converting observed abundance to density. Due to the complexity of implementation, however, both are generally absent from community science databases. The use of structured surveys that include such information, to address the biases in community science data, may allow for unbiased estimates of density and population size.

In this study we evaluated the use of highly structured, professional surveys to address the biases in observed abundance in community science data, while estimating density and population. Specifically, we used models of detection probability built on structured survey data to estimate survey and location specific detection offsets that were included in community science based density models for 16 songbird species. Our primary objective was to assess whether models of imperfect detection from independently gathered, structured data could be used to adjust community science surveys to produce comparable detectability-adjusted estimates of density and population. We approached this objective with three frameworks that emulate realistic scenarios experienced by researchers and conservationists: (1) a post-hoc implementation of an assumed fixed survey radius that ignores imperfect detection and requires no structured data, (2) an independent, pre-existing source of modeled detection probabilities without access to additional data, and (3) an additional, supplemental, calibration dataset collected specifically to adjust available community science data. This final Calibration framework simulates the collection of structured data, specifically intended to adjust existing community science data, when large, independent, structured datasets are unavailable. Additionally, within the Calibration framework, we investigated the effects of calibration dataset size and data pooling on the degree of bias in estimates of density and population. Throughout, density models from the structured dataset were used as benchmarks to compare community science derived estimates of density and regional population size.

## Methods

### Study area and species

We compiled environmental and avian survey data from Benton and Polk counties, Oregon, USA. These counties are located along the western edge of the Willamette Valley and the eastern slope of the Oregon Coast Mountains. The Willamette Valley is dominated by a patchwork of agricultural land whose primary crops include festucoid grasses (turf seed production) and tree- and vine-borne fruits such as hazelnuts and grapes. Remnant fragments of native oak woodlands are dispersed throughout lower elevations, with the largest patches within two National Wildlife Refuges. The coastal mountains are dominated by moist Douglas-Fir (*Pseudotsuga menziesii*) forest. An active timber industry diversifies the age structure of the landscape. Elevation ranges from 150 to 1248 m.

We selected 16 species of passerine that regularly breed in the study area. The selected species represent a wide range of sample size (number of positive occurrences in the dataset) and sample prevalence (proportion of surveys within the community science data in which the species occurs; Table 1), factors that can influence species distribution model (SDM) performance^19^.

**Table 1:** Descriptive statistics for the 16 study species, including species 4-letter codes, in the structured professional and opportunistic community science (eBird) datasets.

Species	Scientific name	Structured professional dataset			Opportunistic community science dataset				
		Prev.	Obs. Occ.	Ind. Det.	Prev.	Obs. Occ.	Ind. Det.	Largest calibration dataset	Local rarity
Bushtit	*Psaltriparus minimus*	0.03	35	59	0.02	19	61	30	R
Wrentit	*Chamaea fasciata*	0.04	46	56	0.03	35	43	30	R
White-breasted Nuthatch	*Sitta carolinensis*	0.03	29	32	0.03	27	31	30	R
House Wren	*Troglodytes aedon*	0.11	119	149	0.16	169	263	100	U
Pacific Wren	*Troglodytes pacificus*	0.22	238	373	0.15	157	212	250	C
Marsh Wren	*Cistothorus palustris*	0.03	34	64	0.01	15	34	30	R
Swainson's Thrush	*Catharus ustulatus*	0.46	497	736	0.39	416	755	250	C
American Robin	*Turdus migratorius*	0.49	524	754	0.42	440	819	250	C
House Finch	*Haemorhous mexicanus*	0.12	128	164	0.05	54	100	100	U
White-crowned Sparrow	*Zonotrichia leucophrys*	0.23	244	389	0.22	232	438	250	C
Song Sparrow	*Melospiza melodia*	0.47	506	750	0.38	406	582	250	C
Spotted Towhee	*Pipilo maculatus*	0.33	357	478	0.37	399	581	250	C
Orange-crowned Warbler	*Leiothlypis celata*	0.26	281	360	0.28	301	408	250	C
Common Yellowthroat	*Geothlypis trichas*	0.25	269	412	0.25	269	460	250	C
Black-throated Gray Warbler	*Setophaga nigrescens*	0.13	144	182	0.12	124	157	100	U
Lazuli Bunting	*Passerina amoena*	0.09	95	116	0.09	98	133	100	U

For each species, total number of surveys ranged between 1060 and 1073 and was equal between the two datasets. Species names and sequences follow American Ornithological Society^49^. Prev., prevalence; Obs. Occ., number of sites observed occupied; Ind. Det., number of individuals detected. Local rarity within the study area was assigned based on number of occurrences and the largest calibration dataset used within the study: C, common; U, uncommon; R, rare.

### Survey datasets & data processing

We used two sources of wildlife survey data throughout our analyses: a highly-structured, professionally-gathered dataset from the Oregon 2020 project^20^, and an opportunistically gathered, semi-structured, community science dataset from eBird.

#### Structured dataset

**Figure 1:**
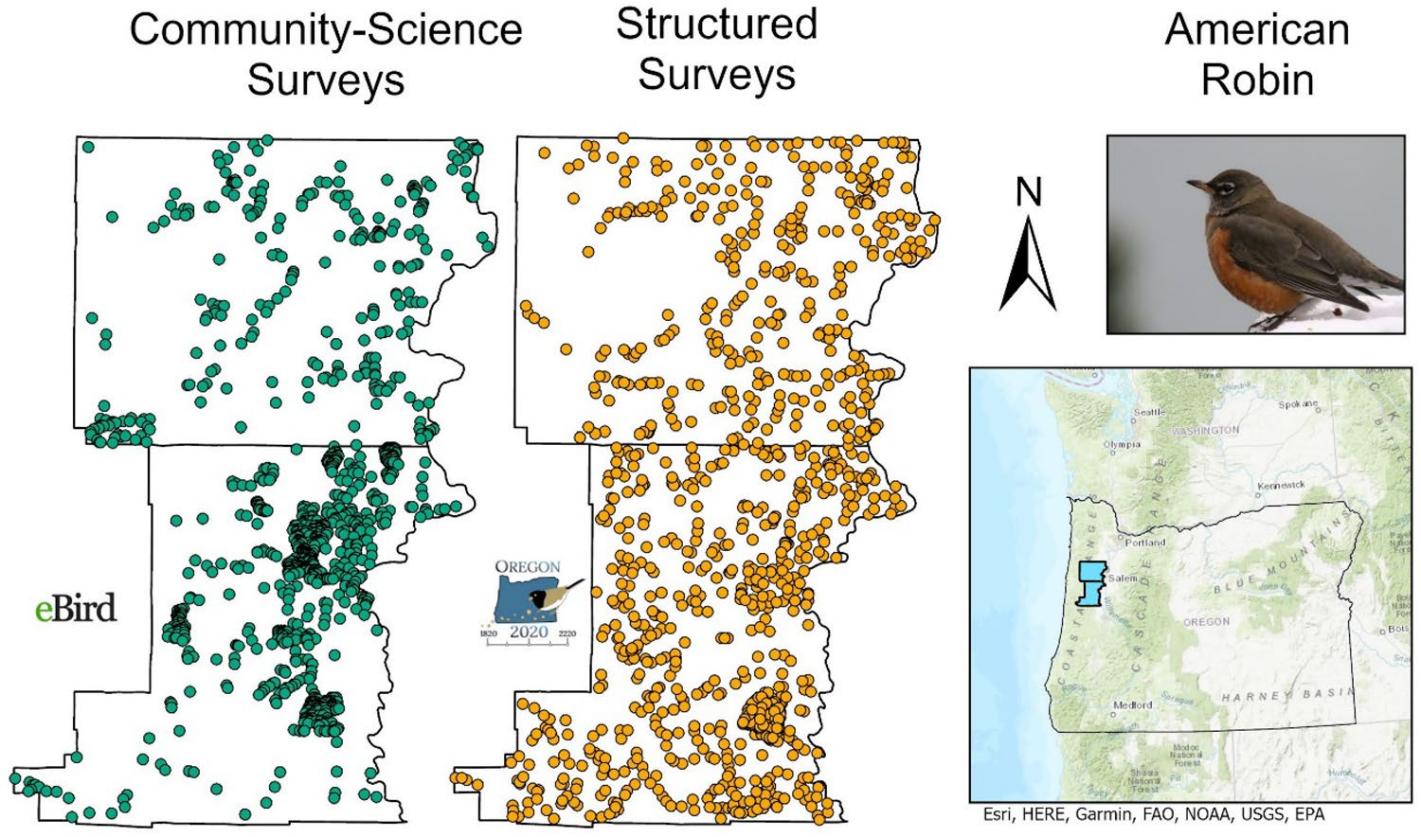
Stringently filtered survey locations in the community science (green) and structured (orange) datasets for American Robin. After stringent filtering and geographic sampling, 1060 community science surveys remained for this species. The structured survey dataset was sampled without replacement to match survey number.

From 2011 to 2013, the Oregon 2020 project conducted 2912 structured bird surveys throughout the study area (Fig. 1)^20^. Trained and experienced observers recorded every bird detected by sight or sound during structured, 5-min, stationary counts. The counts were conducted every 0.8 km along all accessible roads and every 0.2 km off roads within targeted natural habitats. Surveys were conducted during the breeding season (April 30–July 9) from just before sunrise until song activity declined, sometimes up to 7 h after sunrise. To address issues of imperfect detection, time-of-detection^21^ and distance sampling^22^ methods were implemented. For time-of-detection, observers tracked and recorded a detection history for each individual bird through five sequential one-minute intervals. For distance sampling, observers estimated the distance to each individual bird at its initial point of detection and confirmed distances with laser rangefinders. We used Oregon 2020’s highly structured avian surveys in two ways described in depth below. First, these data informed density models using current best practices to estimate densities and populations that serve as benchmarks, against which results from the community science data could be compared. Second, these data were used to model detection probability and calculate offsets to address imperfect detection in the community science dataset. We refer to the Oregon 2020 data as structured data throughout this paper.

For each species, we created benchmark datasets from this structured data. These benchmark datasets were used to inform density models, as described below. Results of community science based models were compared against these benchmark estimates, which were intended to represent current best practices in density modeling. To create benchmark datasets, for each species, we randomly sampled the complete structured dataset without replacement to match the sample sizes of the community science datasets described below. This simultaneously created benchmark datasets and independent test data (e.g. the remaining structured data that were not included in the benchmark dataset) for the calculation of AUC. This process also reduced effects of uneven sample sizes on the comparative performance between community science and benchmark datasets as, generally, models with more data perform better.

#### Opportunistic semi-structured dataset

We downloaded complete eBird checklists from the study area, date range, and years matching the Oregon 2020 surveys (version ebd_relNov‐2017). For each species, we created a separate dataset through stringent filtering. We limited our focus to stationary counts so that environmental data could be directly related to eBird checklist locations. We selected personal locations, as they correspond more closely to the exact locations of stationary counts. We restricted counts to seven hours after nautical dawn and durations to 3 to 30 min. We removed any remaining eBird checklists that contained presence information (e.g. “X”) instead of counts, which resulted in slightly variable numbers of checklists among species (number of surveys ranged from 1060 to 1073 across species). Finally, we used geographic sampling to reduce overrepresentation of birds on territories near popular birding locations. To do this, we created a 200 by 200 m grid over the entire study area and randomly selected one checklist from each grid cell, independent of whether the species was detected. Ideally, there would be sufficient community science surveys within the years in which the structured surveys were conducted, but due to the small numbers of eBird checklists remaining after stringent filtering, we expanded our criteria to include eBird data from 2011 to 2017 (Fig. 1). While expanding criteria temporally greatly augments the number of community science surveys available, it assumes a constant distribution, density, and population size, within the timeframe.

### Environmental data

We compiled data from 25 environmental variables previously used to characterize the conditions in Benton and Polk counties for avian SDMs (Table S1)^19,23^. These variables describe topographic, land cover, and forest structure information acquired from freely available raster datasets^24,25^. We used focal statistics in ArcGIS Pro to calculate percent land cover at five spatial scales shown to be relevant to birds: 75 m, 165 m, 315 m, 615 m, and 1215 m radii from cell centers^19,23,26,27^. We used focal statistics to calculate the mean values for all topographic and forest structure variables at the same spatial extents.

### Frameworks implemented

We implemented three frameworks to mimic the circumstances of real-world researchers and conservationists attempting to model local and regional population sizes from community science data (Table 2). The results of these three community science based models were then compared against benchmark estimates.

**Table 2:** Brief descriptions of the three frameworks implemented in this study.

Framework	Converts abundance to density	Estimates variable survey area	Adjusts for imperfect detection	Includes data pooling	Calibration sample size
Fixed	*Yes*	*No*	*No*	*No*	*NA*
Independent	*Yes*	*Yes*	*Yes*	*No*	*NA*
Calibration	*Yes*	*Yes*	*Yes*	*No*	10
	*Yes*	*Yes*	*Yes*	*No*	30
	*Yes*	*Yes*	*Yes*	*No*	100
	*Yes*	*Yes*	*Yes*	*No*	250
	*Yes*	*Yes*	*Yes*	*Yes*	10
	*Yes*	*Yes*	*Yes*	*Yes*	30
	*Yes*	*Yes*	*Yes*	*Yes*	100
	*Yes*	*Yes*	*Yes*	*Yes*	250

Each framework adjusts community science bird survey data to allow for density estimation. Only the Fixed framework adjusts surveys without the explicit estimation of detection probability and survey area. The Calibration framework was run with and without data pooling in density models to investigate the influence of data pooling and sample sizes on density estimates. See methods for more in-depth descriptions of frameworks.

#### Fixed framework

This framework represents a scenario in which no independent source of distance sampling surveys or detection functions are available and the decision is made to assume a fixed survey radius for all opportunistic community science surveys. The fixed framework assumes perfect detection (i.e., does not account for imperfect detection) within the defined survey area. It is important to note that this survey area is not a part of the field methods employed during surveys, but is defined at the stage of modeling. In contrast to the frameworks described below, where structured data are used to adjust observed abundance for imperfect detection in community science surveys, no structured data are used in, or required for, the Fixed framework. Since this framework uses a constant survey radius across species, resulting “density” estimates are directly related to observed, or unadjusted abundance. For this framework, we converted observed abundance from community science counts to densities using a fixed 200 m survey radius. We chose 200 m because it is a common distance within which most individuals of our set of species and many North American landbird species could be detected by sound (Table S10).

#### Independent framework

This framework represents a scenario in which an independent source of detection functions are available, but the distance sampling surveys used to inform those detection functions are not available. In this framework, the decision is made to use detection functions from another source to calculate offsets that account for imperfect detection and area surveyed without the option for data pooling. This framework could be particularly valuable as researchers and practitioners would not need to conduct structured surveys, but could apply models of detection probability from independent sources to account for imperfect detection in local semi-structured community science data. The use of this framework is now possible, and will likely increase with the growing availability of such models^28^. For this framework, we used the complete structured dataset (2912 surveys) to model detection probability and estimate survey-specific detection offsets. We included detection offsets in density models built on community science surveys.

#### Calibration framework

This framework represents a scenario in which no independent source of distance sampling surveys or detection functions are available and the decision is made to collect supplemental distance sampling surveys with which to model detection probability. As large, pre-existing, structured datasets are rare, this scenario is commonly encountered by conservationists looking to estimate local density and population size with community science data. In this framework, we used subsets of the benchmark datasets to model detection probability and estimate survey-specific detection offsets that account for area surveyed. Within this framework, we examined the effects of two pertinent factors for this scenario: sample size within the calibration dataset, and pooling of calibration and community science data. We created calibration datasets with a range of sample sizes to investigate the degree of survey effort necessary to effectively address bias in community science data. We implemented the calibration framework with and without the pooling of calibration and community science datasets, used in density models. Data pooling of even small calibration datasets may decrease bias of results by ensuring that some of the data included in density models experienced the exact detection processes present in modeled detection probabilities. In this way we investigated the influence sample size and data pooling on the efficacy of calibration datasets.

To create calibration datasets, we randomly sampled benchmark datasets without replacement, until the desired number of surveys with at least one detection of the species reached 10, 30, 100, and 250 occurrences (herein referred to as sample size). Sampling was performed separately within each iteration of the analysis, so calibration datasets were not identical. We used the number of surveys with at least one detection instead of the number of surveys overall, as rarer species might not be detected in a random sample of all surveys, making offset calculation impossible. We used the number of surveys with at least one detection instead of the number of individuals detected to increase the potential environmental variability incorporated in the calculation of offsets (i.e., if one site had 10 individuals and no other points were selected there would be no variation in environmental variables). Due to the low prevalence of some species, only eight species had sufficient detections for inclusion in the largest (N = 250 detections) calibration dataset (Table 1).

### Zero-inflated density models

**Figure 2:**
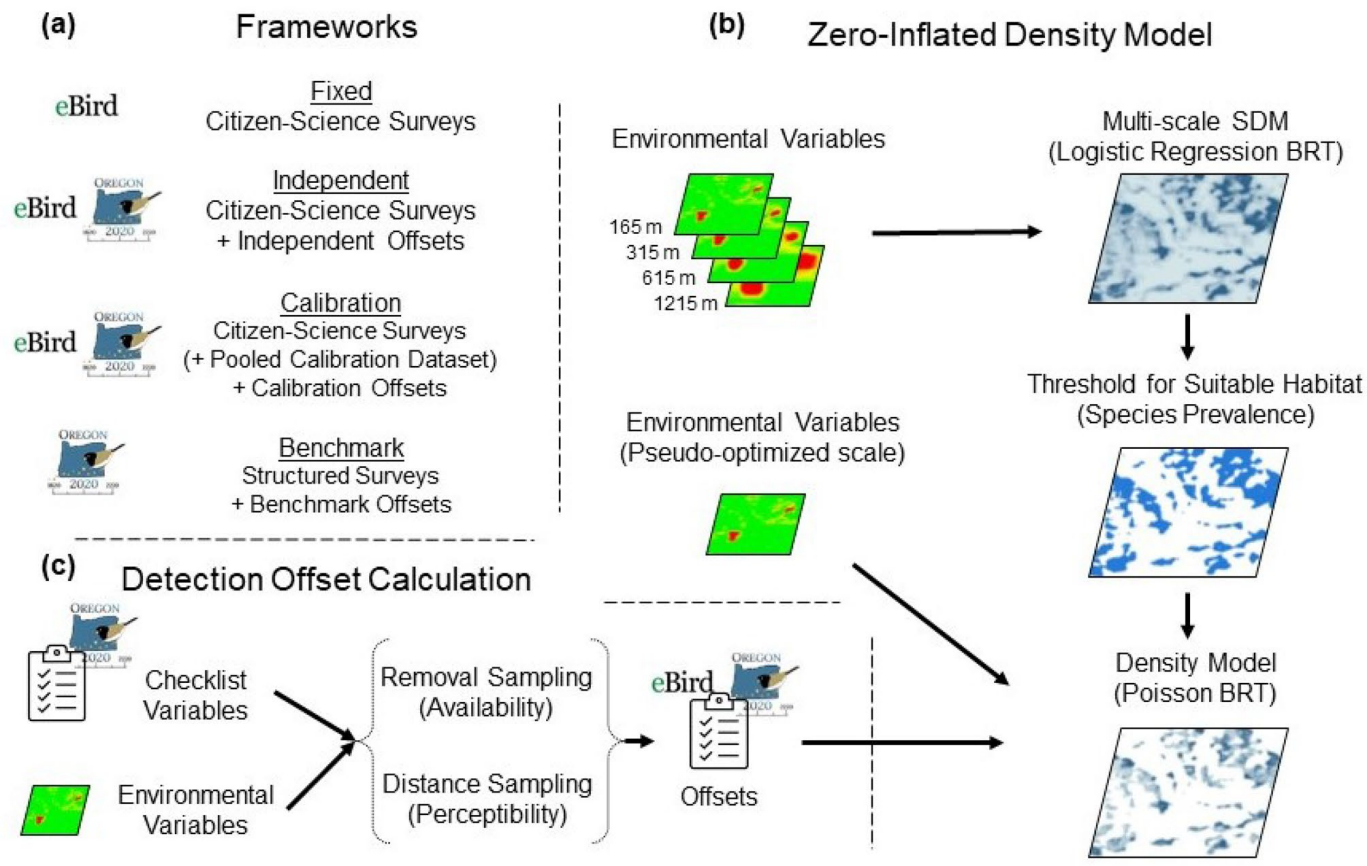
Workflow for analyses including (**a**) the frameworks and datasets, (**b**) the zero-inflated density modeling method, and (**c**) the calculation of detection probability offsets used within density models. The fixed framework incorporates no offsets and assumes a constant area surveyed of 200 m and perfect detection.

For each species and each framework we ran zero-inflated boosted regression tree (BRT) density models (Fig. 2). Generally, zero-inflated BRTs are a three-step process that includes fitting an SDM (logistic BRT) to estimate probability of occurrence, converting probability of occurrence to suitable and unsuitable habitat with a threshold, and fitting a Poisson BRT to estimate abundance within suitable habitat^11^. We modified this method by adding an intermediate step, in which offsets for detection probability that are calculated from structured survey data are included in Poisson BRTs to convert resulting abundance estimates to density (Fig. 2).

#### Zero-inflation

For each species, we fit SDMs with logistic regression BRTs^29^. We set tree complexity to 3, bag fraction to 0.75, and optimized the learning rate so that the optimal number of trees fell between 1000 and 5000. We used a tenfold cross-validation method to construct boosted regression trees and used a multi-scale SDM framework in which we included all environmental variables at all radii^23^. To evaluate models we calculated AUC with the independent test dataset. We then used the sample prevalence of a species within its dataset as the threshold to transform continuous habitat suitability (or probability of occurrence) to binomial suitable and unsuitable habitat^23,30^. We restricted counts used in Poisson BRTs to those occurring in suitable habitat. This first step of the zero-inflated BRT reduces excess zeroes and the influence of counts in unsuitable habitat prior to modeling abundance^11^.

#### Detection offset calculation

In the Independent and Calibration frameworks, detection offsets for community science counts were calculated from detection models built on surveys from the structured dataset, using the QPAD method^31^. No offsets were included in the Fixed framework. Before building models of detection probability, we restricted either the full structured dataset (Independent framework), or the calibration dataset (Calibration framework) to habitat predicted to be suitable for each species, which allowed for the estimation of detection probability within suitable habitat. Imperfect detection is comprised of two components: availability and perceptibility^32^. We ran removal^33^ and distance sampling^22^ model sets for each species to estimate availability and perceptibility, respectively. For removal models, we reduced our time-of-detection data, which included detection histories at each interval within the five-minute count, to removal data by recording the first interval of detection for each individual (i.e. the removal interval). In removal model sets we included combinations of Julian date, time of day (minutes since dawn), and quadratic terms and compared models with AICc.

For distance sampling model sets, we first included distance to the nearest river and distance to the nearest highway (sources of noise) as explanatory variables. We included quadratic terms, log-transformed values, and combinations of distance to river and distance to nearest highway in models. We compared these models to the null with AICc and perpetuated the structure of the top AICc model. We included canopy cover, percent high and medium density urban land cover, and percent total urban land cover, as well as combinations of canopy cover and each of the two urban land cover variables in the subsequent model set. We characterized all land cover covariates in distance models as the mean value within a 75-m radius from cell centers. For all distance models we used 50 m distance bins for distances up to 200 m and included a final bin of all observations over 200 m in distance. The unlimited distance inherent in opportunistic community-science checklists (i.e., observers do not use truncation distances) necessitates an unlimited distance framework^31^. As there is no finite truncation distance, the area sampled is effectively infinite, and estimation of density over an infinite area is impossible. We therefore estimated the effective detection radius (EDR), the radius where the estimated number of individuals missed within the EDR (e.g. not detected) equals the number of individuals detected outside of the EDR, to estimate the effective area sampled. We used the top AICc removal and distance sampling models to calculate offsets (i.e. correction factors) at each survey location^31^. Offsets were calculated as the product of the estimated perceptibility, availability, and effective area sampled. By definition, the perceptibility within the effective area sampled is set to 1.

#### Density model

In the Independent and Calibration frameworks, detection offsets were included within Poisson BRTs to convert resulting abundances to densities. In the Fixed framework, no offsets were included as area surveyed was assumed to be constant and detection probability was assumed to be 1. We set tree complexity to 3, bag fraction to 0.75, and optimized learning rates so that the optimal number of trees fell between 1000 and 5000. To avoid overfitting, we included only pseudo-scale optimized environmental variables previously found to be influential for each species in Poisson BRTs^19,23^. To assess the predictive performance of models, we calculated predictive correlation with the independent test dataset as the correlation between the predicted count at a site derived from estimated densities and offsets, and the observed count. Population estimates were derived from estimated densities. Due to stochasticity involved in the BRT algorithm, and the random sampling of calibration datasets, we ran ten iterations of the above process for each dataset (e.g. each species × dataset combination). These ten iterations were used to assess variability in the results. Zero-inflated density models were run with the *dismo*, *gbm*, and *QPAD* packages in R (version 4.0.3)^31,34–36^.

### Quantifying comparative performance

While we highlight species-specific results below, we were most interested in overarching patterns in comparative performance of each framework’s density models. We therefore converted estimates of each endpoint (AUC, area of suitable habitat, mean density, and population) to a percent of the species-specific benchmark estimate. For each endpoint, benchmarks were calculated as the species-specific median value of the benchmark’s ten iterations. We divided estimates from individual iterations within each framework by these species-specific benchmark values. To reduce the influence of outliers, within our results, we report medians for all summary statistics.

## Results

Stringent filtering reduced 12,572 community science checklists to between 1060 and 1073 (depending on the species) once all criteria were applied (91% reduction; Fig. S1). In benchmark datasets, sample prevalence ranged from 0.03 for Bushtit, Marsh Wren, and White-breasted Nuthatch, to 0.49 for American Robin. In community science datasets, prevalence followed a similar pattern and ranged from 0.01 for Marsh Wren to 0.42 for American Robin (Table 1). Due to low prevalence in some species, only eight species had sufficient detections to create the largest calibration dataset (Table 1).

**Figure 3:**
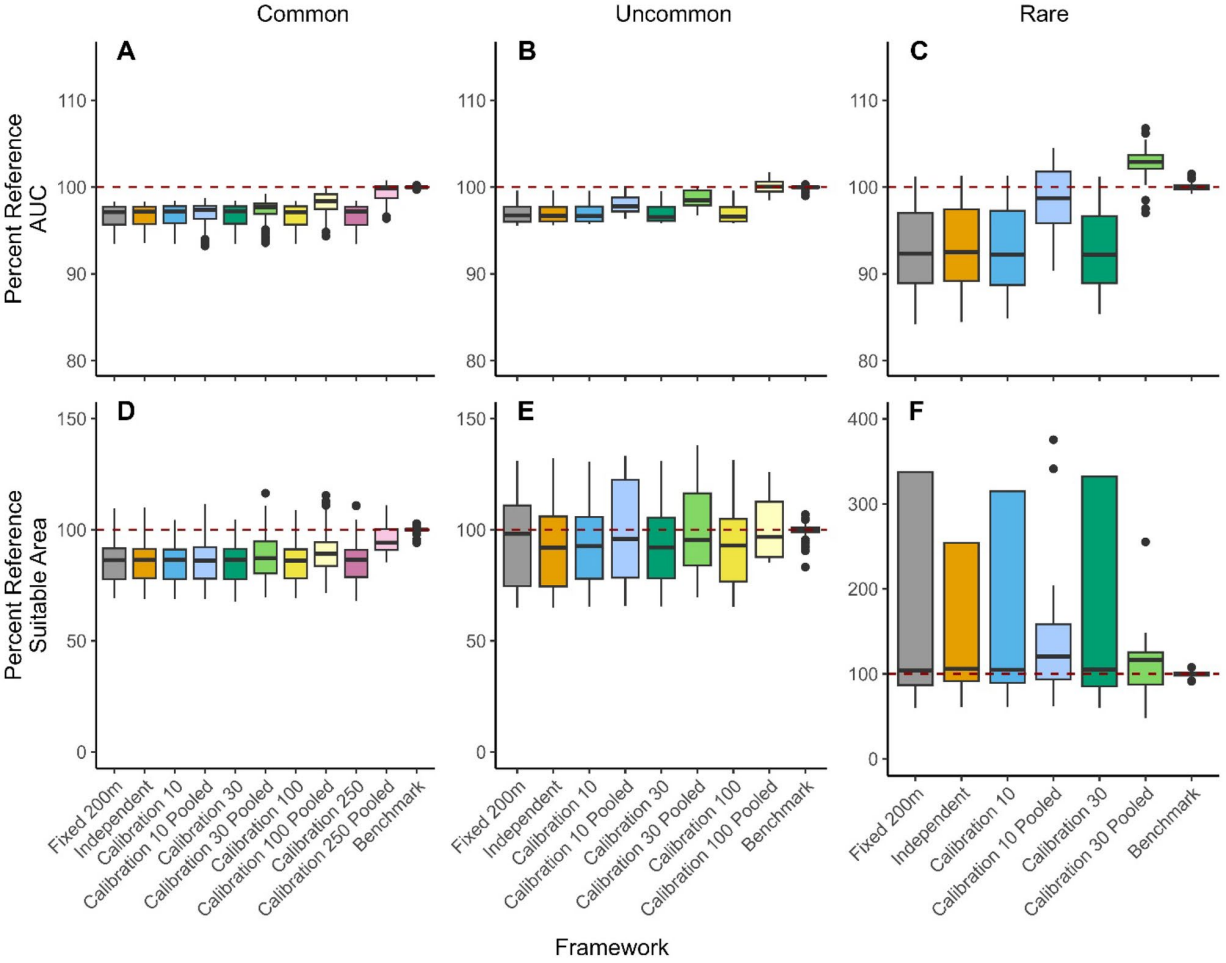
Results from the zero-inflation portion of density models for each framework, including AUC (**A**, **B**, and **C**) and estimated area of suitable habitat (**D**, **E**, and **F**), compared against a best-practices reference (benchmark). To allow for summarization across species, for each species, the results of the ten iterations within a framework were adjusted to the percentage of the median species-specific reference value. Results are divided into species that are common (**A** and **D**; 8 species), uncommon (**B** and **E**; 4 species), and rare (**C** and **F**; 4 species) within our study area as rarer species had insufficient data for the use of larger calibration datasets (Table 1).

SDMs built on community science data generated similar AUCs to benchmarks (Fig. 3). The median AUC from community science SDMs within our zero-inflated BRTs was 0.77 across species, averaging 97% of benchmark values. Median suitable area estimated from community science data was biased marginally low across frameworks (91% of benchmarks). While accuracy of estimates was high for most species, estimates of suitable area for Marsh Wren were a median of ten times higher than benchmarks (Fig. S2). This bias in suitable area for Marsh Wrens was dramatically reduced to 142% of the benchmark by data pooling within the 30-occurrence calibration dataset. Within zero-inflated BRTs, AUC and estimated suitable area are calculated before offsets of detection probability are incorporated. Therefore, across frameworks, in the absence of data pooling, AUC and estimated suitable area remained constant. When data were pooled in the Calibration framework, precision and accuracy of AUC and estimated suitable area increased with calibration dataset size, a pattern especially evident in the eight most common species, which had sufficient detections to create larger calibration datasets (Fig. 3). In species with lower prevalence, pooling of even small calibration datasets had a large influence on AUC. The variance of AUC and estimated suitable area was higher in species with lower prevalence and lower numbers of detections.

**Figure 4:**
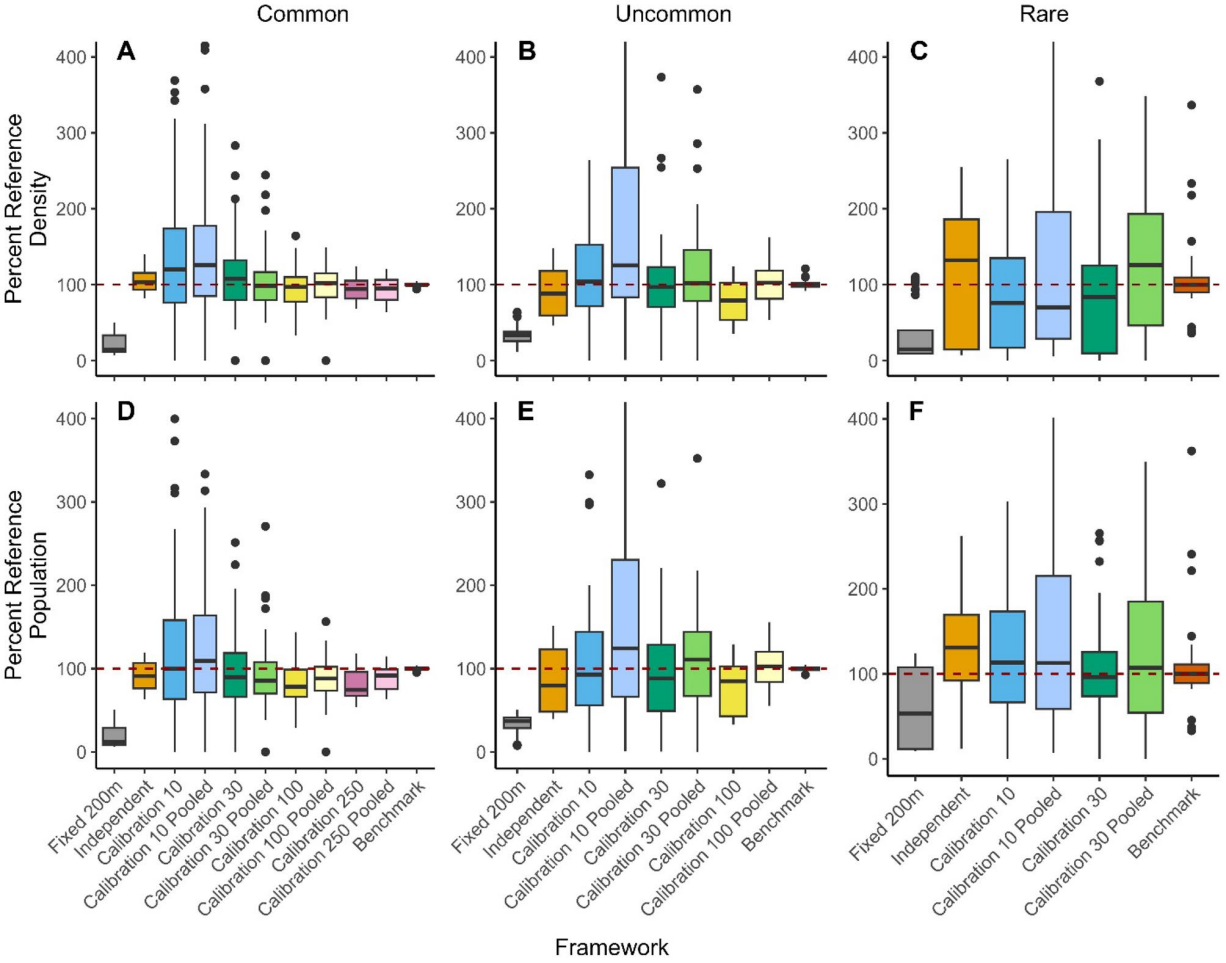
Results from the density portion of density models for each framework, including mean density (**A**, **B**, and **C**) and estimated population (**D**, **E**, and **F**), compared against a best-practices reference (benchmark). To allow for summarization across species, for each species, the results of the ten iterations within a framework were adjusted to the percentage of the median species-specific reference value. Results are divided into species that are common (**A** and **D**; 8 species), uncommon (**B** and **E**; 4 species), and rare (**C** and **F**; 4 species) within our study area as rarer species had insufficient data for the use of larger calibration datasets (Table 1).

Density estimated from community science data was relatively unbiased with a median of 95% of the benchmark values. Precision of density estimates increased with increasing size of the calibration datasets, particularly in sample sizes of 100 and 250 occurrences (Fig. 4). While unbiased across most frameworks, estimated density was biased extremely low in the Fixed framework, with a median of 17% of the benchmark values. Also, while density estimates from the other frameworks were unbiased for most species, for Marsh Wren densities were biased extremely low, with a median of 11% of the benchmark values (Table S6, Fig. S2). Density estimates of House Finch and Black-throated Gray Warbler were also biased low, with 52% and 68% of the benchmark estimate, respectively. Sample sizes implemented in the Calibration frameworks were robust to random variation above N = 30 but substantial variability was apparent at the lowest sample size of N = 10 (Table S7).

Overall, population sizes estimated from community science data were biased low, with a median of 87% of the benchmark values (Fig. 4). Similar to density, population estimates from the Fixed framework were biased extremely low, with a median of 21% of the benchmark estimates. With the exception of rare species, the precision of population estimates across the Calibration frameworks improved with calibration dataset size, with greater improvements in the presence of data-pooling. With data pooling, increased calibration dataset size generally decreased bias (i.e., estimates were closer to benchmarks). In contrast, without data pooling, greater negative bias was introduced with increased calibration dataset size (Fig. 4). Population estimates for House Finch and Black-throated Gray Warbler were biased low, and increases in calibration dataset size, even in data pooling frameworks, did not always result in improved estimates (Table S8, Fig. S2).

## Discussion

We found that even small subsets of structured surveys can be used to address detection bias in free and broadly available community science bird survey data, allowing for the reliable estimation of density and population. The ability to reduce detection bias in community science data, which typically lack the necessary information to account for imperfect detection, while simultaneously estimating an effective survey area, greatly amplifies their conservation value. The substantial bias in our Fixed framework, which lacks adjustments for imperfect detection, emphasizes the risk of estimating populations while taking observed abundance at face value. While this Fixed framework could be greatly improved by using species-specific values, such as maximum detection distance, resulting estimates would remain biased low if detection probability within these distances is ignored^37^. While the bias of community science derived density and population estimates were greatly reduced in both the Calibration and Independent frameworks, we advise a degree of caution when using such methods as the accuracy of estimates were species-specific.

The application of detection functions from the full structured dataset to calculate detection offsets in community science based density models (e.g., the Independent framework), resulted in reliable estimates of density and population for most species. As detection functions with which to calculate these offsets are now available for over 300 landbird species across North America, the use of this Independent framework will likely grow^28^. The species-specific bias of density and population estimates in our study, however, indicate that care must be taken in the use of structured surveys to adjust community science data. Increasing the similarity between structured and community science datasets through stringent filtering, increases the performance of SDMs^10^, and is likely an essential first step in reducing bias in density and population estimates.

We increased alignment of important survey characteristics through stringent filtering, based on count duration, time of day, and locational precision. As our models involve predicting distributions based on habitat characteristics around count locations, community science data must be limited to those surveys using stationary protocols with reliable location information. In eBird, many checklists contributed by birders are traveling counts or stationary counts associated with Hotspot locations. Use of either for our models adds noise and muddles the relationship between observed counts and habitat information. Restricting eBird data to stationary counts at “personal locations” is critical to fine-scale modeling as it reduces much of the locational noise inherent in checklists using other types of protocol (e.g., traveling or incidental) and location (e.g., Hotspot). The use of complete checklists is likewise essential as this allows us to infer absences in checklists without abundances for the species recorded. Geographic sampling or spatial subsampling reduces geographic bias by removing large numbers of counts from popularly surveyed areas. While this may be an important step, it can greatly reduce sample sizes. Here, for example, even with a relatively fine grain of 200 m, geographic sampling reduced the number of opportunistic checklists in our study by around 50 percent (Fig. S1). Geographic bias can have relatively minor impacts on distribution modeling, indicating that geographic sampling may not be strictly necessary^8^. As we did not rerun our models without geographic sampling, we cannot speak to its impacts on our results. Skipping this step, however, might greatly increase sample sizes for rarer species (discussed more below). Although excising the remaining data from analyses greatly reduces sample sizes (Fig. S1), community science datasets are often large enough that sufficient data remain to justify such filtering.

Even with stringent filtering, density and population estimates were biased high in our Independent framework for some species, and low for others. Using models of detection probability, built on structured data, to adjust community science counts, assumes that the detection processes in structured and community science surveys do not differ. For the most part, this seems a reasonable assumption, as important factors such as habitat, extraneous noise, and time-of-day likely impact observers similarly and can be accounted for in stringent filtering and survey-specific detection offsets^31,38,39^. Differences in observer-specific detection probability, however, are not included in these models. For some species, such as American Robin, Lazuli Bunting, Common Yellowthroat, and White-crowned Sparrow, this assumption appeared to be met, as estimates from the Independent and Calibration frameworks matched benchmarks well. For abundant and conspicuous species such as these, models of detection probability from a previously existing source or a supplementary calibration dataset can be used to effectively estimate spatially explicit densities and populations.

For other species, however, including Pacific Wren, Orange-crowned Warbler, Song Sparrow, Spotted Towhee, Swainson’s Thrush, and Wrentit, population estimates from Independent and Calibration frameworks were biased low. This bias can likely be attributed to a violation of the assumption that detection probabilities between structured and community science surveys do not differ. In these cases, higher detection probability in structured surveys would lead to lower community science based population estimates. Heterogeneity in the discrepancies of observed abundance between professional and community science counts tend to be species-and-observer-specific^18^. Whereas observed abundances in community science counts may be accurate for some species, they tend to be biased low for others. In the case of the songbirds listed here, the proportion of detections that are purely auditory could be quite high. These species tend to sing from cover and visual detection can be difficult. On average, counts from community scientists may be more accurate with species detected visually, than aurally. Masking of auditory cues, and the additional effort required to differentiate multiple vocalizing individuals of the same species, may depress count values from community scientists. While there may be little difference in the number of detections between novice and experienced observers for conspicuous and easy-to-identify species, observer expertise is strongly correlated with observed counts in stationary surveys for species that are more difficult to identify^40^. Data pooling of calibration datasets can reduce the bias of estimates and is an important step when discrepancies in detection probability exist. Truly integrated models that allow for the explicit estimation of observer-specific detection probabilities would further address this assumption^41^.

Understanding the reasons for discrepancies in detection probability between structured and community science datasets would greatly increase our confidence in these methods. While eBird’s checklist calibration index, which uses species accumulation curves to account for observer differences in species detection, improves SDM performance^42,43^, no index currently exists to account for differences in the reliability of species counts. Such an index would differ from general detection probabilities as it would need to address common observer-specific behaviors, such as rounding of observed counts, recording numbers from memory well after surveys have ended, and reductions in effort in the detection of subsequent individuals following the initial detection of a species. The development of such an index may greatly reduce the bias of community science based density and population estimates. The choice of when and where to begin a survey also introduces bias in opportunistic community science data if the detection of birds or specific species motivates observers to begin surveys. Databases such as eBird, for example, likely include few surveys where no individuals are detected and many surveys where charismatic or vagrant species are detected. Data pooling of calibration datasets may help to address biases associated with choice of survey initiation.

Large benchmark datasets may not exist for all species in all locations, and conducting large numbers of surveys to create one can be prohibitively expensive. We therefore evaluated the efficacy of collecting smaller supplementary datasets with which to model detection probability and adjust community science data in our Calibration framework. We found that supplementary calibration datasets with as few as ten surveys in which the target species was detected, could produce unbiased community science based estimates of density and population. Combined with the large bias in the one framework where detection probability was ignored (e.g., Fixed Framework), these results strongly suggest that any community science based estimates of density and population should incorporate the explicit estimation of detection probability, even if very few structured surveys can be conducted. If small calibration datasets can be used effectively, financial and temporal limitations may pose much less of a barrier. While bias was low in small calibration datasets, precision of estimates was greatly improved with increasing calibration dataset size, whether or not calibration data were pooled with community science data. As bias in this framework increased with calibration dataset size for some species, researchers should default to data pooling calibration datasets with community science data. The sample size required to produce precise and unbiased estimates is case-specific, but precision of estimates were greatly improved with 30 or more checklists in which target species were detected.

### Small sample size & additional considerations

Small sample sizes in less common species, such as Bushtit, White-breasted Nuthatch, and Marsh Wren, led to some additional challenges in density modeling. False positives, for example, have a very strong influence when sample sizes are low. Marsh Wren is a habitat specialist, only found in marshes, a rare habitat in the study area. Without data pooling, models predicted extremely large areas of suitable habitat and very low densities throughout. The habitat suitability without data pooling was unambiguously incorrect. Inaccurate species distribution models may be due to two primary factors. First, there may be false positives in the community science data. Given the small sample size in this species, any false positives outside of a marsh could have large impacts on an algorithm's ability to differentiate between suitable and unsuitable habitat. Second, there may be true positives in small marshes not accurately identified by satellite imagery. From a modelling perspective, this would present the same issues as false positives. Data pooling greatly increased the accuracy of habitat suitability models for this species, especially when calibration datasets included at least 30 surveys with positive detections.

While estimates of suitable habitat were improved with data pooling, densities within areas of suitable habitat were biased low. There may be at least three contributing factors to biased densities. First, observers may not be visiting marshes when this species is most vocal, and reported abundances may be lower. Second, as this species is primarily identified by sound, community scientists may have lower detection probabilities as some observers may not know how to identify the vocalizations. Third, this species occurs at high densities. At high densities, singing individuals may mask one another, leading to inaccurate counts in community science surveys when effort isn’t put into accurately deciphering the number vocalizing. One of the strengths of community science data is that its large quantity can overwhelm a lower per datum information of structured data^6^. The eBird database continues to grow, and practitioners using community science data can increase sample size by increasing the geographic or temporal scope of the surveys incorporated. For example, we used seven years of data for a single population estimate for each species. Using data from fewer years and larger areas may be more suited to those interested in assessing changes in population size through time. Had we increased the geographic breadth of our study, distribution and density estimates for Marsh Wren may have been much improved. Alternatively, had we chosen to forgo geographic sampling in our study area, rare and geographically restricted marsh habitats would have been far more highly represented as these sites tend to be popular with birders and therefore contain far more opportunistic community science surveys.

On lands without public access, allowing community scientists access can increase data in desired locations without the costs associated with wildlife monitoring. Actively inviting community scientists to conduct surveys, year-round or on restricted dates, could further augment desired data while simultaneously engaging the public. Active participation in local conservation can improve conservation actions and help address current biodiversity issues^44,45^. When conducting supplemental calibration surveys, encompassing the environmental variability of the geographic area of interest and the variability in survey level characteristics (e.g. time of day or day of year), is important to minimize extrapolation. Although geographic overlap is the clear ideal for a structured dataset, it may not be essential if overlap in environmental space is sufficient.

### Suitable area & threshold selection

For many species, the estimated area of suitable habitat was biased low compared to benchmarks. While discrepancies in detection probabilities likely play a role, differences in the breadth of geographic and environmental sampling may also be a contributing factor. Machine learning algorithms such as BRTs can include erroneous relationships between environmental variables and occurrence or abundance when there is insufficient variation to inform models across environmental space. Greater geographic clustering in community science data can lead to less variation in environmental variables, which can lead to lower estimated area of suitable habitat (Fig. 1). In the presence of clustering in community science surveys, collection and pooling of additional data from structured surveys collected in habitats or locations unsurveyed by the community science data may address this issue.

It is also important to note that we used species prevalence within opportunistic community science checklists as thresholds when converting continuous habitat suitability to binary suitable habitat. We chose prevalence because it is species-specific and easily calculated from community science data. Many options for thresholding exist, and we did not specifically examine the sensitivity of our modeling to threshold selection. Area of suitable habitat based on binary suitability is sensitive to threshold selection and thresholding results from species distribution models can impact conservation prioritization^46,47^. This may therefore be an important area of continued research and threshold selection should be considered when using these methods. Alternative methods of thresholding can easily be incorporated into the modeling methods used here. As the modeling methods employed here only use data from within suitable habitat to model density, population estimates in this study are somewhat robust to threshold selection. For a given species, lower thresholds result in larger areas of predicted suitable habitat. Generally, these larger areas incorporate a greater number and proportion of low abundance counts, which reduce the model predicted densities. Higher thresholds result in smaller areas of predicted suitable habitat. Generally, these smaller areas include a greater proportion of high abundance counts, resulting in higher predicted densities.

## Conclusion

Although opportunistic community science data can be used to produce high performing species distribution models^10,17^, moving beyond predicted distributions to densities greatly benefits conservation and management^48^. Density and derived population estimates allow conservationists to assess the system’s current state, set conservation goals, and evaluate the success of management actions. Biodiversity monitoring is expensive, currently making up a significant portion of total conservation costs^49^. Many local conservation organizations report fiscal barriers to the monitoring necessary to assess the success of their conservation actions^4^. Combined with freely available, remotely sensed environmental data, community science data provide a cost-effective method of monitoring wildlife populations^50^. These methods will be used moving forward^28^, so understanding their strengths and limitations is essential.

In this study, we used independent structured survey data to model detection functions and calculate offsets for community science surveys. Reliable estimation of density and population size with community science data would greatly increase their conservation value. We found that although independent detection functions could be used to produce accurate estimates for some species, there was relatively high bias in others. The collection of supplemental calibration survey data with which to model detection probability was similarly accurate for some species and biased for others. Data pooling of calibration datasets greatly decreased bias, and should be implemented in conjunction with stringent filtering and geographic sampling, where sample sizes are sufficient.

### Supplementary Information

Supplementary Information.

## Data Availability

All data used in this study are available at: https://figshare.com/articles/dataset/R_Scripts_and_data_used_for_peer-reviewed_paper_/24723756.
