# Supplementary material for: Supplemental structured surveys and pre-existing detection models improve fine-scale density and population estimation with opportunistic community science data
Source: Sci Rep. 2024 May 14;14:11070. doi: 10.1038/s41598-024-61582-6 (PMC11094051; doi:10.1038/s41598-024-61582-6)
Supplement: Supplementary file 1 — Supplementary Information. [file 41598_2024_61582_MOESM1_ESM.docx]

**Title:** Maximizing the value of opportunistic citizen science: evaluating the use of supplemental structured surveys in density and population estimation

**Supplementary Tables and Figures**

**Supplementary Table S1.** Environmental variables used in models of distribution and density. Data were gathered from freely available raster datasets ^2,3^ and were previously used to model species distributions ^4,5^. Table is copied from Hallman & Robinson ^5^.

| **Source** | **Type** | **Statistic** | **Variable Name** |
| --- | --- | --- | --- |
| National Gap Analysis Project | Land Cover | Percent | Temperate Forest |
|  |  |  | North Pacific Oak Woodland |
|  |  |  | Temperate, Grassland, Meadow & Shrubland |
|  |  |  | Western North American Freshwater Wet Meadow & Marsh |
|  |  |  | Willamette Valley Upland Prairie and Savanna |
|  |  |  | Willamette Valley Wet Prairie |
|  |  |  | Pasture/Hay |
|  |  |  | Orchards Vineyards and Other High Structure Agriculture |
|  |  |  | Cultivated Cropland |
|  |  |  | Developed, Open Space |
|  |  |  | Developed, Low Intensity |
|  |  |  | Developed, Medium Intensity |
|  |  |  | Developed, High Intensity |
| Gradient Nearest Neighbor | Forest Structure & Composition | Mean | Canopy Cover of Conifers |
|  |  |  | Canopy Cover of Hardwoods |
|  |  |  | Total Canopy Cover |
|  |  |  | Quercus Canopy Cover |
|  |  |  | Shrub Cover |
|  |  |  | Diameter Diversity Index |
|  |  |  | Density of Live Trees >= 2.5 cm dbh |
|  |  |  | Basal Area of Live Trees >= 2.5 cm dbh |
| Oregon Spatial Data Library | Topographic |  | Elevation |
|  |  |  | Slope |
|  |  |  | Compound Topographic Index |
|  | Distance |  | Distance to River |

**Supplementary Table S2.** Predictive performance of species distribution models (SDMs) measured as area under the curve (AUC) for 16 species of passerine from each of the ten citizen science frameworks and the best-practices benchmark. Values represent the median from ten iterations of each framework. Missing values are from species with low prevalence (see Table S1).

| **Species** | **Fixed 200m** | **Independent** | **Calibration 10** | **Calibration 10 Pooled** | **Calibration 30** | **Calibration 30 Pooled** | **Calibration 100** | **Calibration 100 Pooled** | **Calibration 250** | **Calibration 250 Pooled** | **Benchmark** |
| --- | --- | --- | --- | --- | --- | --- | --- | --- | --- | --- | --- |
| Bushtit | 0.68 | 0.68 | 0.68 | 0.72 | 0.67 | 0.75 | . | . | . | . | 0.71 |
| Wrentit | 0.64 | 0.64 | 0.64 | 0.71 | 0.64 | 0.75 | . | . | . | . | 0.74 |
| White-breasted Nuthatch | 0.72 | 0.72 | 0.72 | 0.77 | 0.72 | 0.82 | . | . | . | . | 0.80 |
| House Wren | 0.73 | 0.73 | 0.73 | 0.73 | 0.73 | 0.74 | 0.73 | 0.75 | . | . | 0.75 |
| Pacific Wren | 0.91 | 0.91 | 0.91 | 0.92 | 0.91 | 0.92 | 0.91 | 0.93 | 0.91 | 0.93 | 0.93 |
| Marsh Wren | 0.93 | 0.93 | 0.93 | 0.94 | 0.93 | 0.95 | . | . | . | . | 0.92 |
| Swainson's Thrush | 0.79 | 0.79 | 0.79 | 0.79 | 0.79 | 0.79 | 0.79 | 0.80 | 0.79 | 0.80 | 0.81 |
| American Robin | 0.72 | 0.72 | 0.72 | 0.72 | 0.72 | 0.72 | 0.72 | 0.73 | 0.72 | 0.74 | 0.76 |
| House Finch | 0.88 | 0.88 | 0.88 | 0.88 | 0.88 | 0.88 | 0.88 | 0.88 | . | . | 0.88 |
| White-crowned Sparrow | 0.77 | 0.77 | 0.77 | 0.77 | 0.77 | 0.78 | 0.77 | 0.80 | 0.77 | 0.82 | 0.82 |
| Song Sparrow | 0.77 | 0.77 | 0.77 | 0.77 | 0.77 | 0.77 | 0.77 | 0.78 | 0.77 | 0.78 | 0.79 |
| Spotted Towhee | 0.74 | 0.74 | 0.74 | 0.74 | 0.74 | 0.74 | 0.74 | 0.75 | 0.74 | 0.76 | 0.76 |
| Orange-crowned Warbler | 0.77 | 0.77 | 0.77 | 0.78 | 0.77 | 0.78 | 0.77 | 0.79 | 0.77 | 0.80 | 0.80 |
| Common Yellowthroat | 0.78 | 0.78 | 0.78 | 0.78 | 0.78 | 0.78 | 0.78 | 0.79 | 0.78 | 0.80 | 0.80 |
| Black-throated Gray Warbler | 0.78 | 0.78 | 0.78 | 0.79 | 0.78 | 0.80 | 0.78 | 0.81 | . | . | 0.81 |
| Lazuli Bunting | 0.76 | 0.76 | 0.76 | 0.78 | 0.77 | 0.79 | 0.76 | 0.81 | . | . | 0.80 |

**Supplementary Table S3.** Standard deviation of predictive performance of species distribution models (SDMs) measured as area under the curve (AUC) for 16 species of passerine from each of the ten citizen science frameworks and the best-practices benchmark. Values represent the standard deviation from ten iterations of each framework.

| **Species** | **Fixed 200m** | **Independent** | **Calibration 10** | **Calibration 10 Pooled** | **Calibration 30** | **Calibration 30 Pooled** | **Calibration 100** | **Calibration 100 Pooled** | **Calibration 250** | **Calibration 250 Pooled** | **Benchmark** |
| --- | --- | --- | --- | --- | --- | --- | --- | --- | --- | --- | --- |
| Bushtit | 0.00 | 0.00 | 0.00 | 0.02 | 0.00 | 0.01 |  |  |  |  | 0.01 |
| Wrentit | 0.01 | 0.01 | 0.01 | 0.02 | 0.01 | 0.01 |  |  |  |  | 0.00 |
| White-breasted Nuthatch | 0.00 | 0.00 | 0.00 | 0.01 | 0.00 | 0.00 |  |  |  |  | 0.00 |
| House Wren | 0.00 | 0.00 | 0.00 | 0.00 | 0.00 | 0.00 | 0.00 | 0.00 |  |  | 0.00 |
| Pacific Wren | 0.00 | 0.00 | 0.00 | 0.00 | 0.00 | 0.00 | 0.00 | 0.00 | 0.00 | 0.00 | 0.00 |
| Marsh Wren | 0.00 | 0.00 | 0.00 | 0.01 | 0.00 | 0.00 |  |  |  |  | 0.00 |
| Swainson's Thrush | 0.00 | 0.00 | 0.00 | 0.00 | 0.00 | 0.00 | 0.00 | 0.00 | 0.00 | 0.00 | 0.00 |
| American Robin | 0.00 | 0.00 | 0.00 | 0.00 | 0.00 | 0.00 | 0.00 | 0.00 | 0.00 | 0.00 | 0.00 |
| House Finch | 0.00 | 0.00 | 0.00 | 0.00 | 0.00 | 0.00 | 0.00 | 0.00 |  |  | 0.00 |
| White-crowned Sparrow | 0.00 | 0.00 | 0.00 | 0.00 | 0.00 | 0.00 | 0.00 | 0.00 | 0.00 | 0.00 | 0.00 |
| Song Sparrow | 0.00 | 0.00 | 0.00 | 0.00 | 0.00 | 0.00 | 0.00 | 0.00 | 0.00 | 0.00 | 0.00 |
| Spotted Towhee | 0.00 | 0.00 | 0.00 | 0.00 | 0.00 | 0.00 | 0.00 | 0.00 | 0.00 | 0.00 | 0.00 |
| Orange-crowned Warbler | 0.00 | 0.00 | 0.00 | 0.00 | 0.00 | 0.00 | 0.00 | 0.00 | 0.00 | 0.00 | 0.00 |
| Common Yellowthroat | 0.00 | 0.00 | 0.00 | 0.00 | 0.00 | 0.00 | 0.00 | 0.00 | 0.00 | 0.00 | 0.00 |
| Black-throated Gray Warbler | 0.00 | 0.00 | 0.00 | 0.00 | 0.00 | 0.00 | 0.00 | 0.00 |  |  | 0.00 |
| Lazuli Bunting | 0.00 | 0.00 | 0.00 | 0.01 | 0.00 | 0.01 | 0.00 | 0.00 |  |  | 0.00 |

**Supplementary Table S4.** Area of habitat predicted to be suitable (km^2^) for 16 species of passerine from each of the ten citizen science frameworks and the best-practices benchmark. Values represent the median from ten iterations of each framework.

| **Species** | **Fixed 200m** | **Independent** | **Calibration 10** | **Calibration 10 Pooled** | **Calibration 30** | **Calibration 30 Pooled** | **Calibration 100** | **Calibration 100 Pooled** | **Calibration 250** | **Calibration 250 Pooled** | **Benchmark** |
| --- | --- | --- | --- | --- | --- | --- | --- | --- | --- | --- | --- |
| Bushtit | 314 | 323 | 319 | 335 | 314 | 246 |  |  |  |  | 308 |
| Wrentit | 671 | 681 | 685 | 785 | 685 | 930 |  |  |  |  | 1030 |
| White-breasted Nuthatch | 551 | 550 | 583 | 648 | 558 | 647 |  |  |  |  | 520 |
| House Wren | 838 | 848 | 841 | 854 | 834 | 841 | 847 | 803 |  |  | 660 |
| Pacific Wren | 1116 | 1116 | 1119 | 1147 | 1117 | 1182 | 1123 | 1217 | 1114 | 1375 | 1553 |
| Marsh Wren | 415 | 406 | 499 | 143 | 439 | 50 |  |  |  |  | 35 |
| Swainson's Thrush | 1996 | 1998 | 2002 | 2004 | 2007 | 2034 | 1992 | 2107 | 2008 | 2177 | 2321 |
| American Robin | 1341 | 1336 | 1331 | 1332 | 1338 | 1352 | 1332 | 1403 | 1328 | 1474 | 1463 |
| House Finch | 682 | 685 | 685 | 693 | 686 | 687 | 686 | 650 |  |  | 693 |
| White-crowned Sparrow | 1236 | 1248 | 1245 | 1254 | 1246 | 1312 | 1241 | 1323 | 1234 | 1333 | 1206 |
| Song Sparrow | 1200 | 1199 | 1200 | 1213 | 1195 | 1218 | 1202 | 1238 | 1198 | 1247 | 1319 |
| Spotted Towhee | 977 | 978 | 974 | 969 | 973 | 1010 | 971 | 1021 | 978 | 1101 | 1223 |
| Orange-crowned Warbler | 967 | 937 | 958 | 962 | 934 | 1010 | 964 | 1093 | 901 | 1294 | 1293 |
| Common Yellowthroat | 691 | 693 | 669 | 665 | 683 | 733 | 680 | 651 | 668 | 702 | 766 |
| Black-throated Gray Warbler | 644 | 636 | 642 | 724 | 636 | 800 | 641 | 994 |  |  | 966 |
| Lazuli Bunting | 446 | 444 | 451 | 441 | 454 | 476 | 452 | 470 |  |  | 543 |

**Supplementary Table S5.** Standard deviation of area of habitat predicted to be suitable (km^2^) for 16 species of passerine from each of the ten citizen science frameworks and the best-practices benchmark. Values represent the standard deviation from ten iterations of each framework.

| **Species** | **Fixed 200m** | **Independent** | **Calibration 10** | **Calibration 10 Pooled** | **Calibration 30** | **Calibration 30 Pooled** | **Calibration 100** | **Calibration 100 Pooled** | **Calibration 250** | **Calibration 250 Pooled** | **Benchmark** |
| --- | --- | --- | --- | --- | --- | --- | --- | --- | --- | --- | --- |
| Bushtit | 14 | 14 | 14 | 87 | 22 | 52 |  |  |  |  | 16 |
| Wrentit | 37 | 28 | 32 | 103 | 36 | 136 |  |  |  |  | 29 |
| White-breasted Nuthatch | 34 | 22 | 48 | 95 | 30 | 19 |  |  |  |  | 11 |
| House Wren | 13 | 20 | 13 | 27 | 25 | 45 | 21 | 15 |  |  | 7 |
| Pacific Wren | 15 | 19 | 11 | 47 | 14 | 54 | 13 | 66 | 20 | 22 | 47 |
| Marsh Wren | 24 | 82 | 71 | 100 | 51 | 14 |  |  |  |  | 1 |
| Swainson's Thrush | 10 | 12 | 11 | 29 | 8 | 38 | 11 | 59 | 11 | 35 | 7 |
| American Robin | 10 | 33 | 22 | 46 | 31 | 77 | 25 | 66 | 32 | 41 | 5 |
| House Finch | 4 | 7 | 4 | 85 | 6 | 54 | 4 | 30 |  |  | 54 |
| White-crowned Sparrow | 11 | 16 | 12 | 31 | 13 | 35 | 14 | 53 | 21 | 4 | 9 |
| Song Sparrow | 22 | 4 | 6 | 28 | 5 | 25 | 7 | 45 | 7 | 28 | 6 |
| Spotted Towhee | 8 | 9 | 7 | 17 | 9 | 23 | 6 | 33 | 5 | 32 | 4 |
| Orange-crowned Warbler | 36 | 43 | 37 | 36 | 40 | 51 | 37 | 62 | 47 | 62 | 14 |
| Common Yellowthroat | 73 | 57 | 38 | 68 | 27 | 84 | 56 | 56 | 85 | 19 | 10 |
| Black-throated Gray Warbler | 7 | 5 | 5 | 39 | 4 | 63 | 8 | 59 |  |  | 11 |
| Lazuli Bunting | 58 | 15 | 11 | 85 | 9 | 74 | 16 | 5 |  |  | 14 |

**Supplementary Table S6.** Estimated density per hectare within suitable habitat for 16 species of passerine from each of the ten citizen science frameworks and the best-practices benchmark. Values represent the median from ten iterations of each framework.

| **Species** | **Fixed 200m** | **Independent** | **Calibration 10** | **Calibration 10 Pooled** | **Calibration 30** | **Calibration 30 Pooled** | **Calibration 100** | **Calibration 100 Pooled** | **Calibration 250** | **Calibration 250 Pooled** | **Benchmark** |
| --- | --- | --- | --- | --- | --- | --- | --- | --- | --- | --- | --- |
| Bushtit | 0.04 | 0.75 | 0.52 | 0.78 | 0.59 | 0.78 |  |  |  |  | 0.42 |
| Wrentit | 0.02 | 0.18 | 0.11 | 0.06 | 0.09 | 0.11 |  |  |  |  | 0.09 |
| White-breasted Nuthatch | 0.02 | 0.01 | 0.01 | 0.02 | 0.02 | 0.04 |  |  |  |  | 0.02 |
| House Wren | 0.06 | 0.20 | 0.22 | 0.30 | 0.22 | 0.19 | 0.16 | 0.18 |  |  | 0.18 |
| Pacific Wren | 0.07 | 0.65 | 0.68 | 0.82 | 0.65 | 0.89 | 0.71 | 0.75 | 0.71 | 0.78 | 0.73 |
| Marsh Wren | 0.02 | 0.03 | 0.02 | 0.05 | 0.02 | 0.09 |  |  |  |  | 0.23 |
| Swainson's Thrush | 0.12 | 1.56 | 2.79 | 2.18 | 1.73 | 1.41 | 1.31 | 1.33 | 1.29 | 1.30 | 1.64 |
| American Robin | 0.10 | 0.29 | 0.34 | 0.32 | 0.36 | 0.20 | 0.27 | 0.27 | 0.26 | 0.24 | 0.26 |
| House Finch | 0.04 | 0.06 | 0.07 | 0.09 | 0.05 | 0.09 | 0.05 | 0.11 |  |  | 0.11 |
| White-crowned Sparrow | 0.10 | 0.20 | 0.23 | 0.29 | 0.22 | 0.21 | 0.22 | 0.22 | 0.21 | 0.19 | 0.19 |
| Song Sparrow | 0.08 | 0.56 | 0.54 | 0.86 | 0.61 | 0.67 | 0.49 | 0.51 | 0.50 | 0.53 | 0.66 |
| Spotted Towhee | 0.07 | 0.65 | 0.62 | 0.85 | 0.56 | 0.43 | 0.47 | 0.51 | 0.46 | 0.46 | 0.48 |
| Orange-crowned Warbler | 0.06 | 0.53 | 0.60 | 0.59 | 0.42 | 0.38 | 0.38 | 0.38 | 0.43 | 0.34 | 0.51 |
| Common Yellowthroat | 0.09 | 0.40 | 0.53 | 0.36 | 0.38 | 0.37 | 0.35 | 0.36 | 0.37 | 0.35 | 0.31 |
| Black-throated Gray Warbler | 0.04 | 0.20 | 0.32 | 0.31 | 0.25 | 0.23 | 0.18 | 0.21 |  |  | 0.31 |
| Lazuli Bunting | 0.04 | 0.10 | 0.09 | 0.17 | 0.09 | 0.11 | 0.08 | 0.09 |  |  | 0.08 |

**Supplementary Table S7.** Standard deviation of estimated density per hectare within suitable habitat for 16 species of passerine from each of the ten citizen science frameworks and the best-practices benchmark. Values represent the standard deviation from ten iterations of each framework.

| **Species** | **Fixed 200m** | **Independent** | **Calibration 10** | **Calibration 10 Pooled** | **Calibration 30** | **Calibration 30 Pooled** | **Calibration 100** | **Calibration 100 Pooled** | **Calibration 250** | **Calibration 250 Pooled** | **Benchmark** |
| --- | --- | --- | --- | --- | --- | --- | --- | --- | --- | --- | --- |
| Bushtit | 0.001 | 0.139 | ^1^ | 0.625 | 0.276 | 0.739 |  |  |  |  | 0.399 |
| Wrentit | 0.002 | 0.146 | ^1^ | 1983.405 | 0.095 | 0.057 |  |  |  |  | 0.038 |
| White-breasted Nuthatch | 0.002 | 0.009 | 0.014 | 0.026 | 0.009 | 0.019 |  |  |  |  | 0.004 |
| House Wren | 0.001 | 0.008 | 0.150 | 17.222 | 0.165 | 0.168 | 0.010 | 0.017 |  |  | 0.005 |
| Pacific Wren | 0.002 | 0.014 | 0.307 | 0.340 | 0.326 | 0.182 | 0.095 | 0.046 | 0.045 | 0.017 | 0.013 |
| Marsh Wren | 0.002 | 0.007 | 0.013 | 0.041 | 0.013 | 0.039 |  |  |  |  | 0.025 |
| Swainson's Thrush | 0.001 | 0.021 | ^1^ | 1.561 | 0.316 | 2.370 | 0.367 | 0.325 | 0.054 | 0.123 | 0.030 |
| American Robin | 0.003 | 0.008 | 0.221 | 0.515 | 0.142 | 0.042 | 0.040 | 0.038 | 0.017 | 0.016 | 0.002 |
| House Finch | 0.000 | 0.004 | 0.052 | 0.062 | 0.170 | 0.065 | 0.020 | 0.035 |  |  | 0.010 |
| White-crowned Sparrow | 0.002 | 0.004 | 0.102 | 10.867 | 0.102 | 0.093 | 0.018 | 0.026 | 0.006 | 0.002 | 0.004 |
| Song Sparrow | 0.002 | 0.015 | 0.517 | 245.390 | 0.383 | 0.269 | 0.045 | 0.247 | 0.033 | 0.056 | 0.011 |
| Spotted Towhee | 0.001 | 0.012 | 0.510 | ^1^ | 0.281 | 0.222 | 0.081 | 0.097 | 0.019 | 0.040 | 0.006 |
| Orange-crowned Warbler | 0.002 | 0.011 | 0.467 | 0.561 | 0.176 | 0.176 | 0.082 | 0.055 | 0.018 | 0.021 | 0.012 |
| Common Yellowthroat | 0.007 | 0.025 | 0.248 | 0.211 | 0.107 | 0.142 | 0.075 | 0.034 | 0.031 | 0.013 | 0.011 |
| Black-throated Gray Warbler | 0.002 | 0.016 | 0.839 | 7152.595 | 0.083 | 0.098 | 0.018 | 0.025 |  |  | 0.011 |
| Lazuli Bunting | 0.005 | 0.005 | 0.155 | 0.148 | 0.176 | 0.044 | 0.004 | 0.005 |  |  | 0.002 |

^1^Exceptionally high standard deviation due to extreme outliers in a subset of model iterations.

**Supplementary Table S8.** Estimated population (thousands) within the study area for 16 species of passerine from each of the ten citizen science frameworks and the best-practices benchmark. Values represent the median from ten iterations of each framework.

| **Species** | **Fixed 200m** | **Independent** | **Calibration 10** | **Calibration 10 Pooled** | **Calibration 30** | **Calibration 30 Pooled** | **Calibration 100** | **Calibration 100 Pooled** | **Calibration 250** | **Calibration 250 Pooled** | **Benchmark** |
| --- | --- | --- | --- | --- | --- | --- | --- | --- | --- | --- | --- |
| Bushtit | 1.2 | 23.8 | 16.7 | 34.2 | 16.7 | 19.1 |  |  |  |  | 12.9 |
| Wrentit | 1.4 | 12.6 | 7.5 | 5.0 | 6.5 | 9.7 |  |  |  |  | 9.2 |
| White-breasted Nuthatch | 1.3 | 0.5 | 0.9 | 1.4 | 0.9 | 2.8 |  |  |  |  | 1.2 |
| House Wren | 4.7 | 16.3 | 18.2 | 25.6 | 18.2 | 16.7 | 13.0 | 14.8 |  |  | 11.9 |
| Pacific Wren | 7.8 | 72.3 | 76.1 | 94.6 | 73.8 | 105.1 | 79.5 | 96.5 | 79.2 | 108.7 | 112.8 |
| Marsh Wren | 0.9 | 1.0 | 1.0 | 0.8 | 0.8 | 0.4 |  |  |  |  | 0.8 |
| Swainson's Thrush | 24.3 | 312.1 | 559.4 | 429.8 | 345.4 | 286.5 | 262.3 | 280.9 | 256.4 | 287.3 | 380.7 |
| American Robin | 13.9 | 37.9 | 45.0 | 41.0 | 46.5 | 27.4 | 34.5 | 37.9 | 34.2 | 35.6 | 37.5 |
| House Finch | 2.6 | 3.8 | 4.9 | 6.1 | 3.5 | 5.7 | 3.7 | 7.0 |  |  | 7.4 |
| White-crowned Sparrow | 11.7 | 24.9 | 28.5 | 36.4 | 27.7 | 27.1 | 26.7 | 27.6 | 25.8 | 25.1 | 23.3 |
| Song Sparrow | 9.8 | 66.7 | 65.5 | 102.9 | 72.7 | 80.1 | 58.4 | 62.2 | 60.3 | 65.6 | 87.0 |
| Spotted Towhee | 7.3 | 63.3 | 59.9 | 83.7 | 54.3 | 43.0 | 46.1 | 51.0 | 44.6 | 50.2 | 59.0 |
| Orange-crowned Warbler | 6.0 | 49.6 | 56.2 | 56.9 | 38.3 | 37.2 | 36.2 | 39.9 | 39.3 | 43.8 | 65.9 |
| Common Yellowthroat | 6.1 | 27.4 | 33.6 | 24.2 | 25.8 | 27.0 | 23.7 | 23.4 | 25.0 | 24.3 | 23.9 |
| Black-throated Gray Warbler | 2.4 | 12.6 | 20.8 | 22.3 | 15.8 | 19.2 | 11.2 | 21.6 |  |  | 29.5 |
| Lazuli Bunting | 1.9 | 4.5 | 4.2 | 8.2 | 3.9 | 5.3 | 3.7 | 4.3 |  |  | 4.1 |

**Supplementary Table S9.** Standard deviation of the estimated population within the study area for 16 species of passerine from each of the ten citizen science frameworks and the best-practices benchmark. Values represent the standard deviation from ten iterations of each framework.

| **Species** | **Fixed 200m** | **Independent** | **Calibration 10** | **Calibration 10 Pooled** | **Calibration 30** | **Calibration 30 Pooled** | **Calibration 100** | **Calibration 100 Pooled** | **Calibration 250** | **Calibration 250 Pooled** | **Benchmark** |
| --- | --- | --- | --- | --- | --- | --- | --- | --- | --- | --- | --- |
| Bushtit | 0.03 | 4.28 | ^1^ | 19.12 | 7.74 | 20.06 |  |  |  |  | 13.32 |
| Wrentit | 0.16 | 8.86 | ^1^ | ^1^ | 6.86 | 5.64 |  |  |  |  | 3.84 |
| White-breasted Nuthatch | 0.05 | 0.49 | 0.75 | 1.59 | 0.53 | 1.21 |  |  |  |  | 0.19 |
| House Wren | 0.08 | 0.83 | 12.65 | 1402.39 | 13.32 | 14.98 | 1.02 | 1.50 |  |  | 0.35 |
| Pacific Wren | 0.14 | 0.79 | 33.78 | 41.18 | 35.87 | 22.32 | 10.85 | 6.00 | 5.21 | 2.29 | 1.79 |
| Marsh Wren | 0.10 | 0.29 | 0.65 | 0.33 | 0.44 | 0.19 |  |  |  |  | 0.11 |
| Swainson's Thrush | 0.17 | 4.44 | ^1^ | 318.19 | 63.35 | 480.23 | 73.03 | 70.79 | 11.01 | 28.90 | 6.51 |
| American Robin | 0.46 | 0.73 | 29.15 | 72.27 | 19.30 | 6.08 | 5.32 | 5.42 | 2.27 | 2.47 | 0.33 |
| House Finch | 0.02 | 0.25 | 3.57 | 4.16 | 11.63 | 4.71 | 1.42 | 2.34 |  |  | 0.14 |
| White-crowned Sparrow | 0.13 | 0.29 | 12.67 | 1356.68 | 12.53 | 12.20 | 2.22 | 3.94 | 0.74 | 0.32 | 0.36 |
| Song Sparrow | 0.24 | 1.85 | 62.22 | ^1^ | 45.99 | 33.46 | 5.43 | 30.45 | 3.91 | 7.76 | 1.63 |
| Spotted Towhee | 0.12 | 1.63 | 49.39 | ^1^ | 27.23 | 21.24 | 7.73 | 11.02 | 1.90 | 3.75 | 0.75 |
| Orange-crowned Warbler | 0.29 | 2.96 | 45.23 | 52.10 | 16.95 | 17.18 | 6.84 | 7.67 | 3.33 | 1.91 | 1.32 |
| Common Yellowthroat | 0.18 | 0.90 | 16.93 | 14.43 | 6.83 | 13.32 | 5.14 | 1.99 | 1.41 | 1.33 | 0.63 |
| Black-throated Gray Warbler | 0.15 | 1.01 | 53.04 | ^1^ | 5.24 | 6.74 | 1.17 | 3.15 |  |  | 0.90 |
| Lazuli Bunting | 0.08 | 0.20 | 6.94 | 8.62 | 7.80 | 1.76 | 0.23 | 0.25 |  |  | 0.14 |

^1^Exceptionally high standard deviation due to extreme outliers in a subset of model iterations.

**Supplementary Table S10.** Effective detection radius (EDR) and availability (p) for the structured professional dataset. See methods for information about the variables included in models of imperfect detection.

| **Species** | **Mean EDR** | **Max. EDR** | **Min. EDR** | **Mean p** | **Max. p** | **Min. p** |
| --- | --- | --- | --- | --- | --- | --- |
| Bushtit | 33.10 | 33.10 | 33.10 | 0.36 | 1.00 | 0.00 |
| Wrentit | 82.18 | 152.15 | 12.83 | 0.66 | 0.66 | 0.66 |
| White-breasted Nuthatch | 95.85 | 131.71 | 32.32 | 0.83 | 1.00 | 0.07 |
| House Wren | 95.00 | 95.00 | 95.00 | 0.99 | 1.00 | 0.95 |
| Pacific Wren | 66.01 | 66.01 | 66.01 | 0.97 | 0.99 | 0.91 |
| Marsh Wren | 136.70 | 497.59 | 29.79 | 0.99 | 1.00 | 0.85 |
| Swainson's Thrush | 54.32 | 72.36 | 38.26 | 0.94 | 0.99 | 0.81 |
| American Robin | 131.28 | 196.56 | 64.97 | 0.96 | 0.99 | 0.95 |
| House Finch | 126.79 | 158.02 | 70.26 | 0.88 | 0.95 | 0.76 |
| White-crowned Sparrow | 137.74 | 151.55 | 92.66 | 0.98 | 0.99 | 0.95 |
| Song Sparrow | 81.45 | 111.86 | 39.71 | 0.97 | 0.99 | 0.95 |
| Spotted Towhee | 80.92 | 113.58 | 41.43 | 0.96 | 0.98 | 0.86 |
| Orange-crowned Warbler | 81.45 | 107.63 | 57.32 | 0.94 | 0.97 | 0.66 |
| Common Yellowthroat | 92.75 | 142.70 | 48.59 | 0.94 | 1.00 | 0.88 |
| Black-throated Gray Warbler | 86.69 | 125.61 | 64.28 | 0.92 | 0.97 | 0.87 |
| Lazuli Bunting | 105.45 | 216.95 | 52.69 | 0.99 | 0.99 | 0.99 |


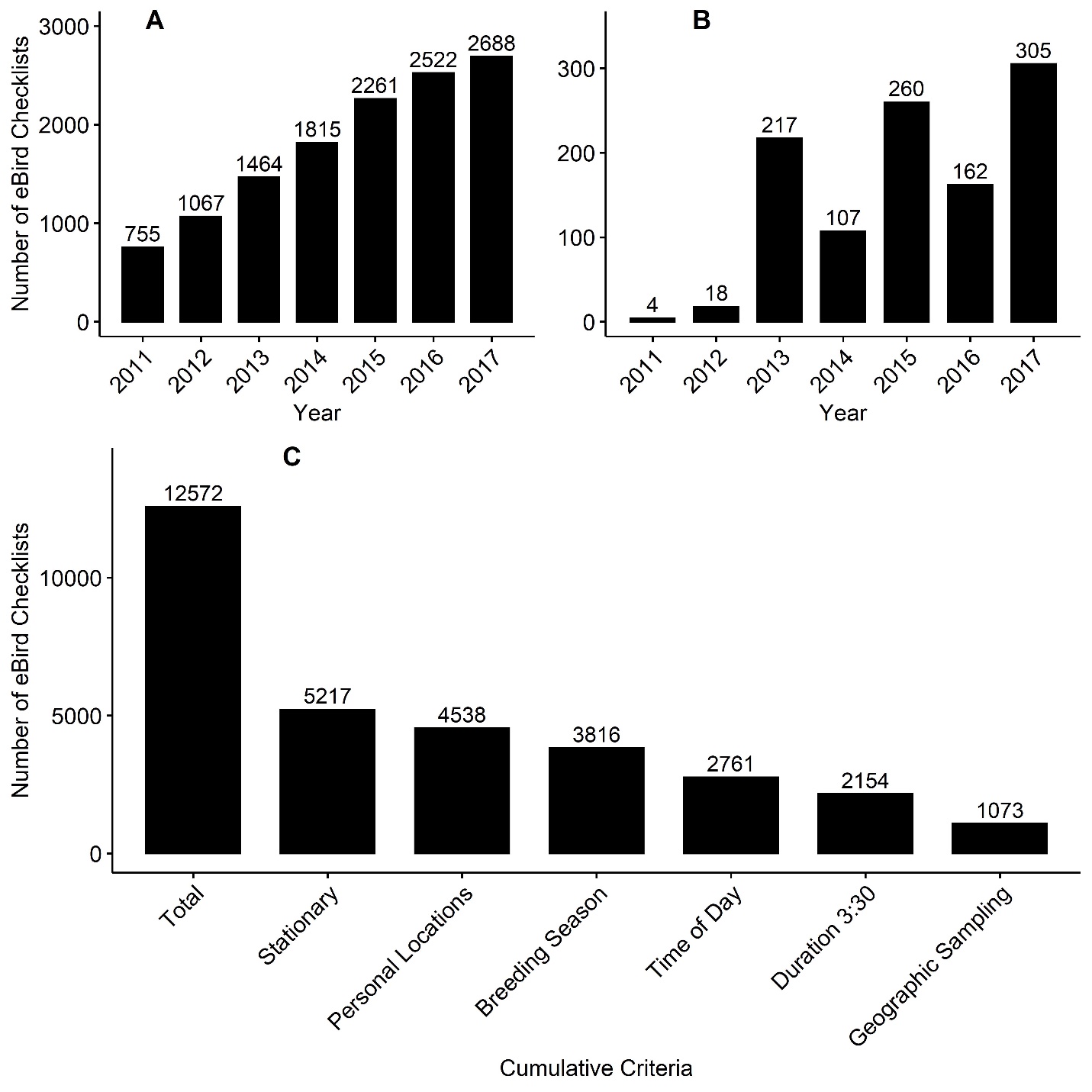


**Supplementary Figure S1.** Counts of opportunistic citizen science checklists (eBird) in study area from 2011 to 2017: (A) total number of checklists by year, (B) total number of checklists meeting criteria by year, and (C) number of checklists over the entire time period remaining for use in our analyses after each cumulative filtering criterion was applied, leading to our final N of 1073 checklists.


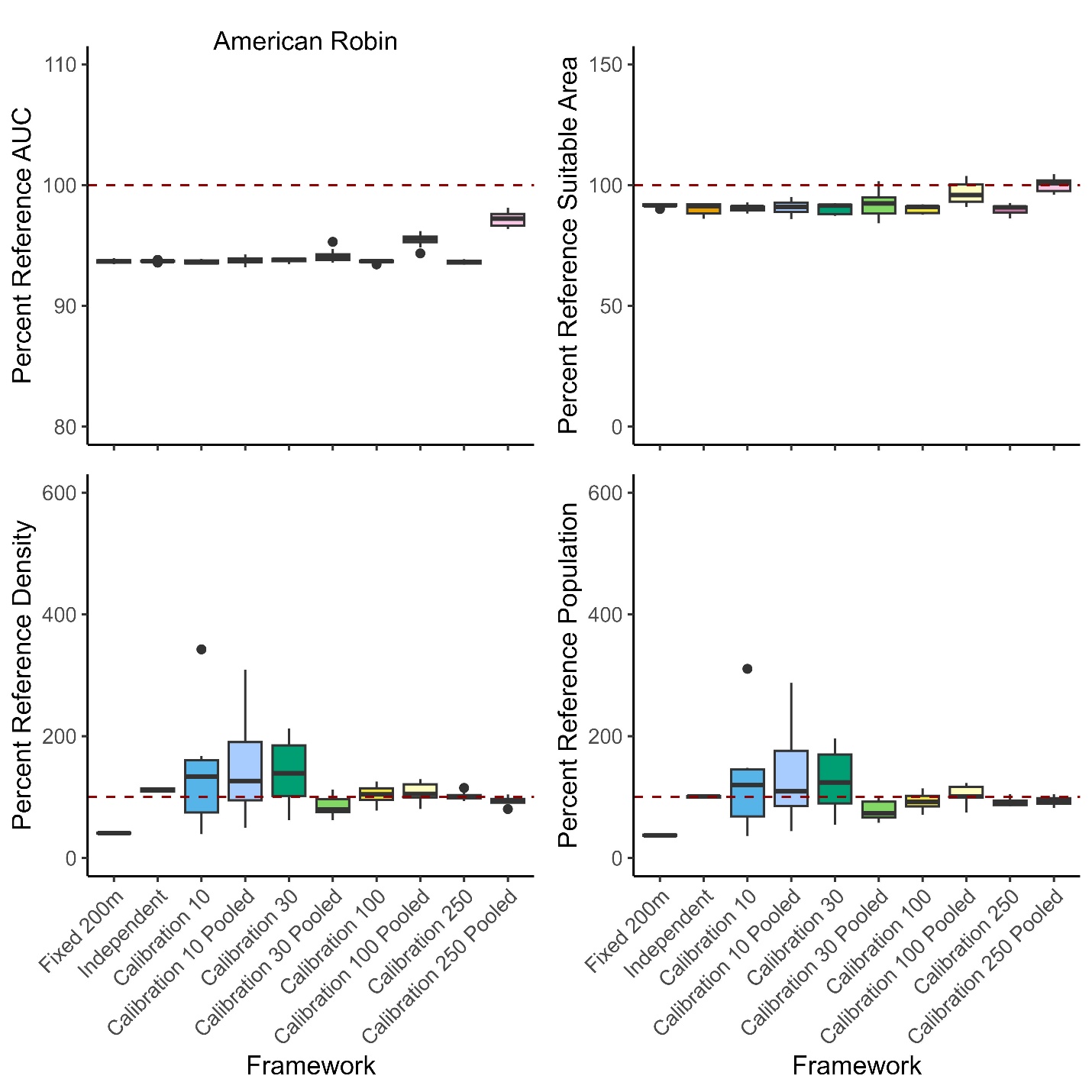


**Supplementary Figure S2.** Performance of all frameworks compared against the reference structured dataset for each of the 16 study species.


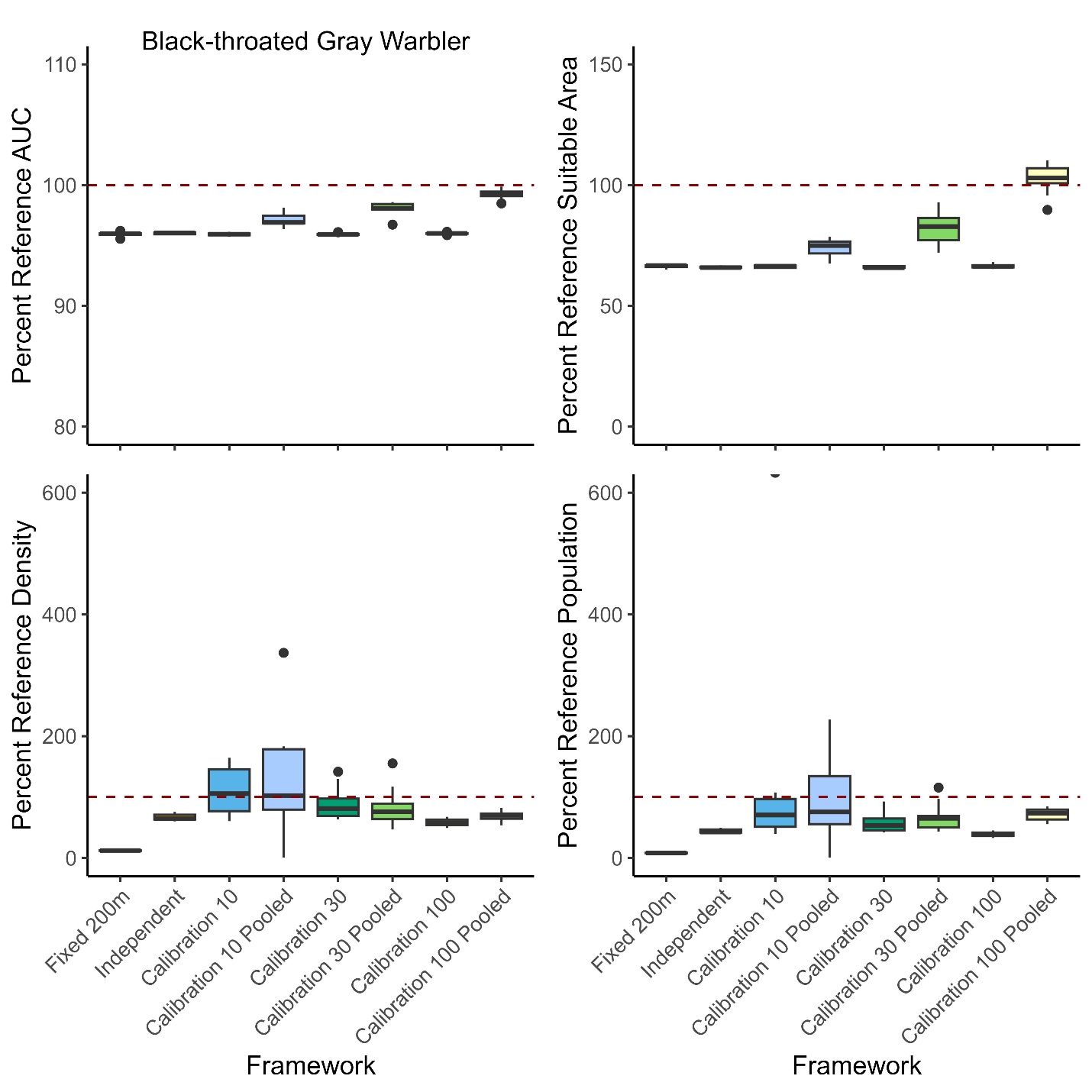


**Supplementary Figure S2.** Continued.


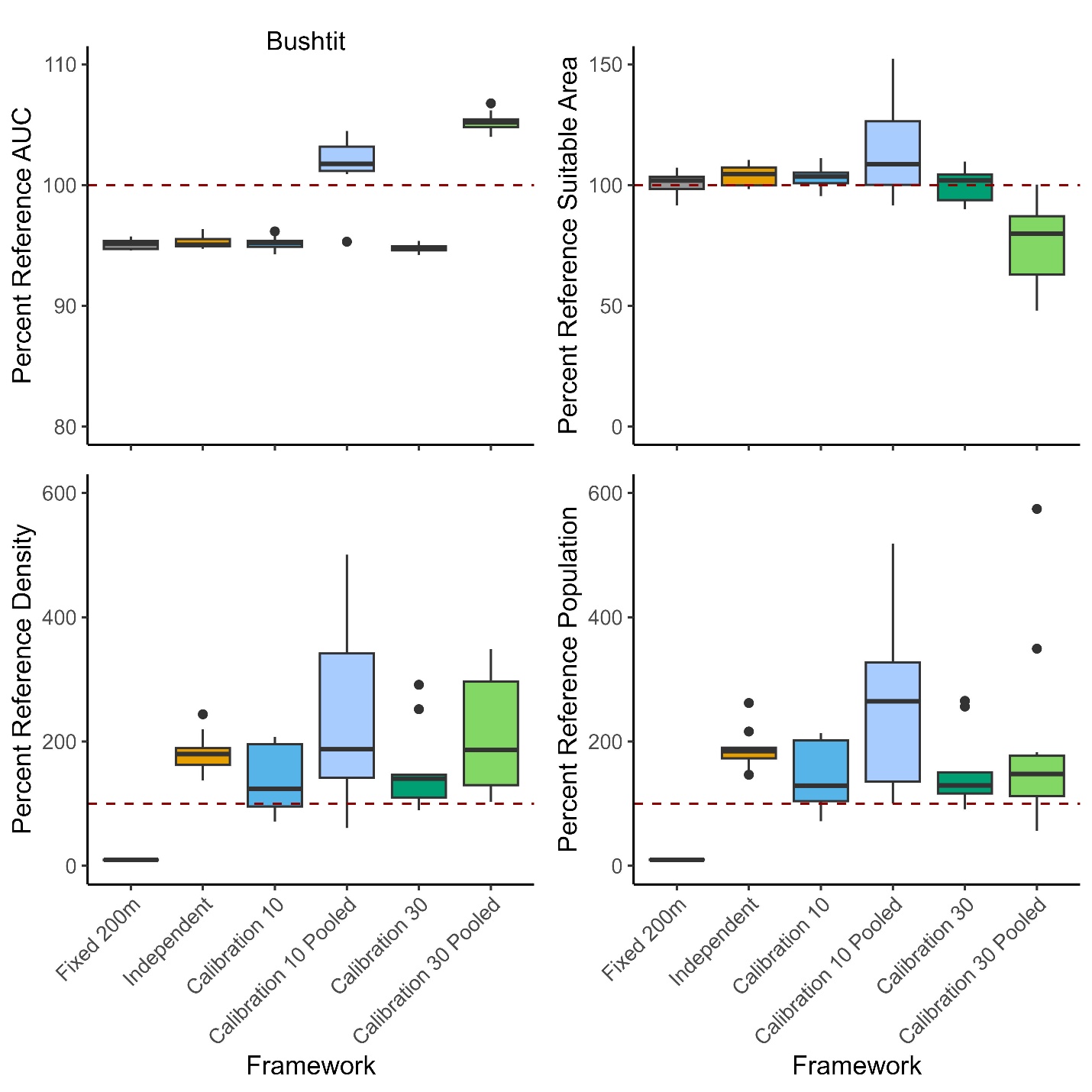


**Supplementary Figure S2.** Continued.


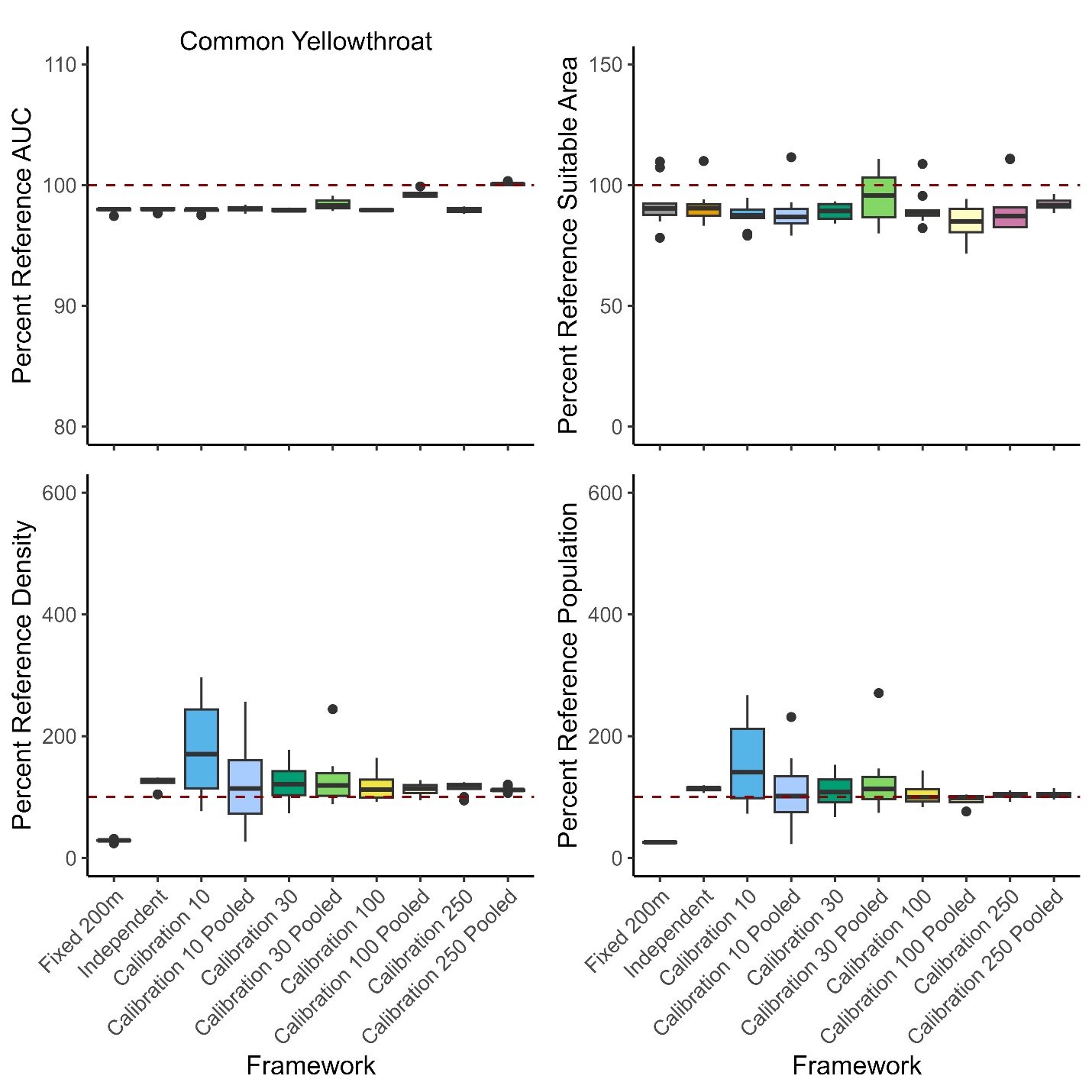


**Supplementary Figure S2.** Continued.


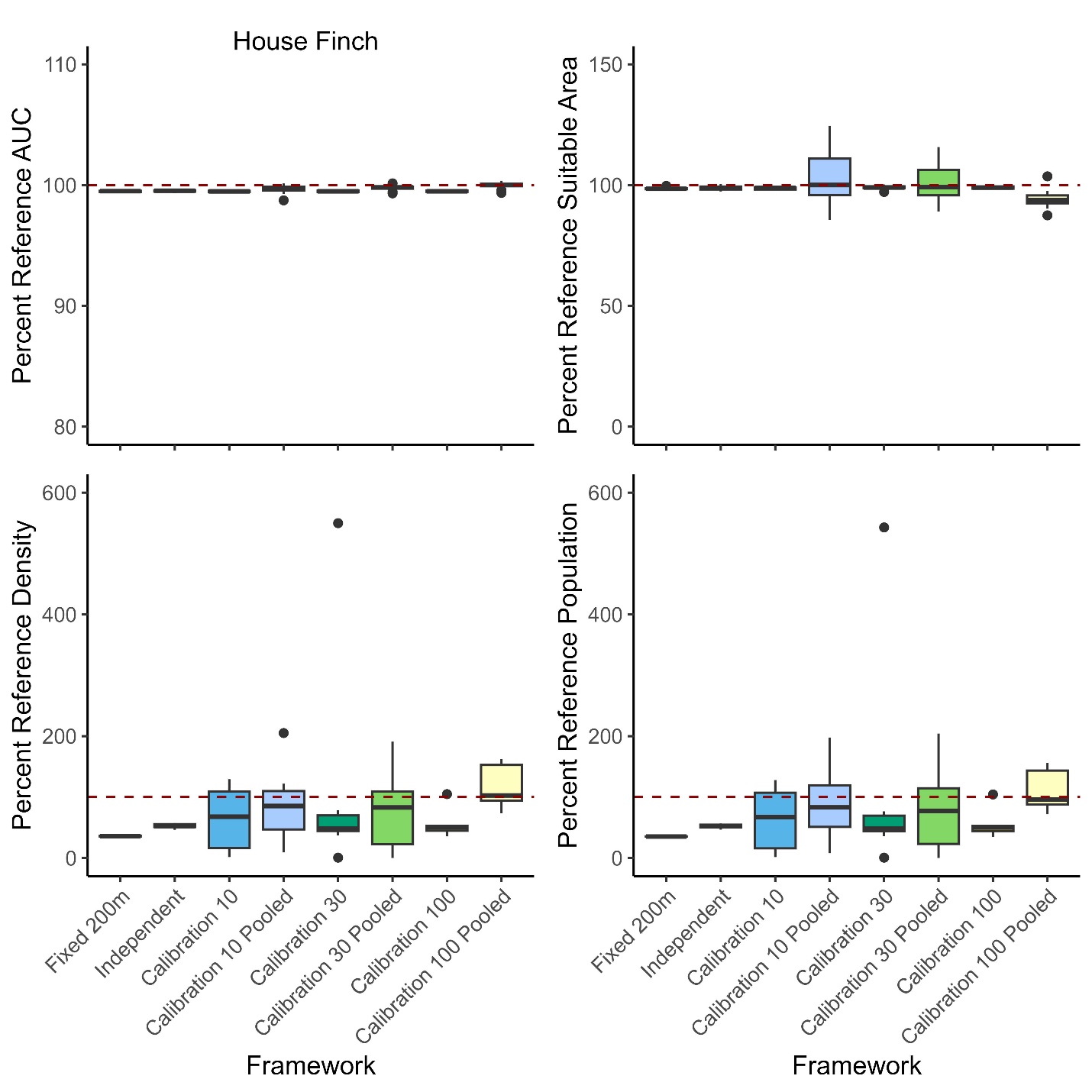


**Supplementary Figure S2.** Continued.


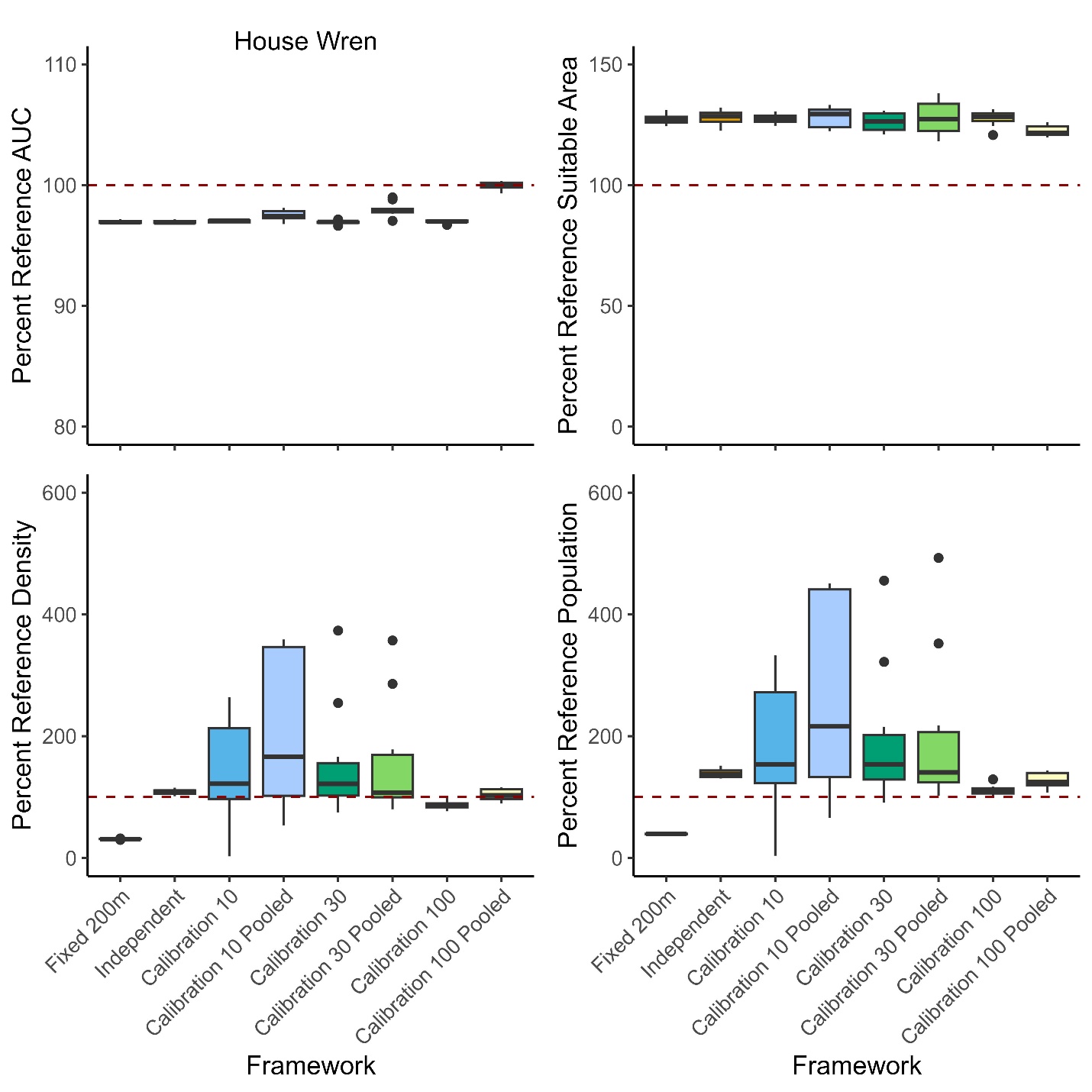


**Supplementary Figure S2.** Continued.


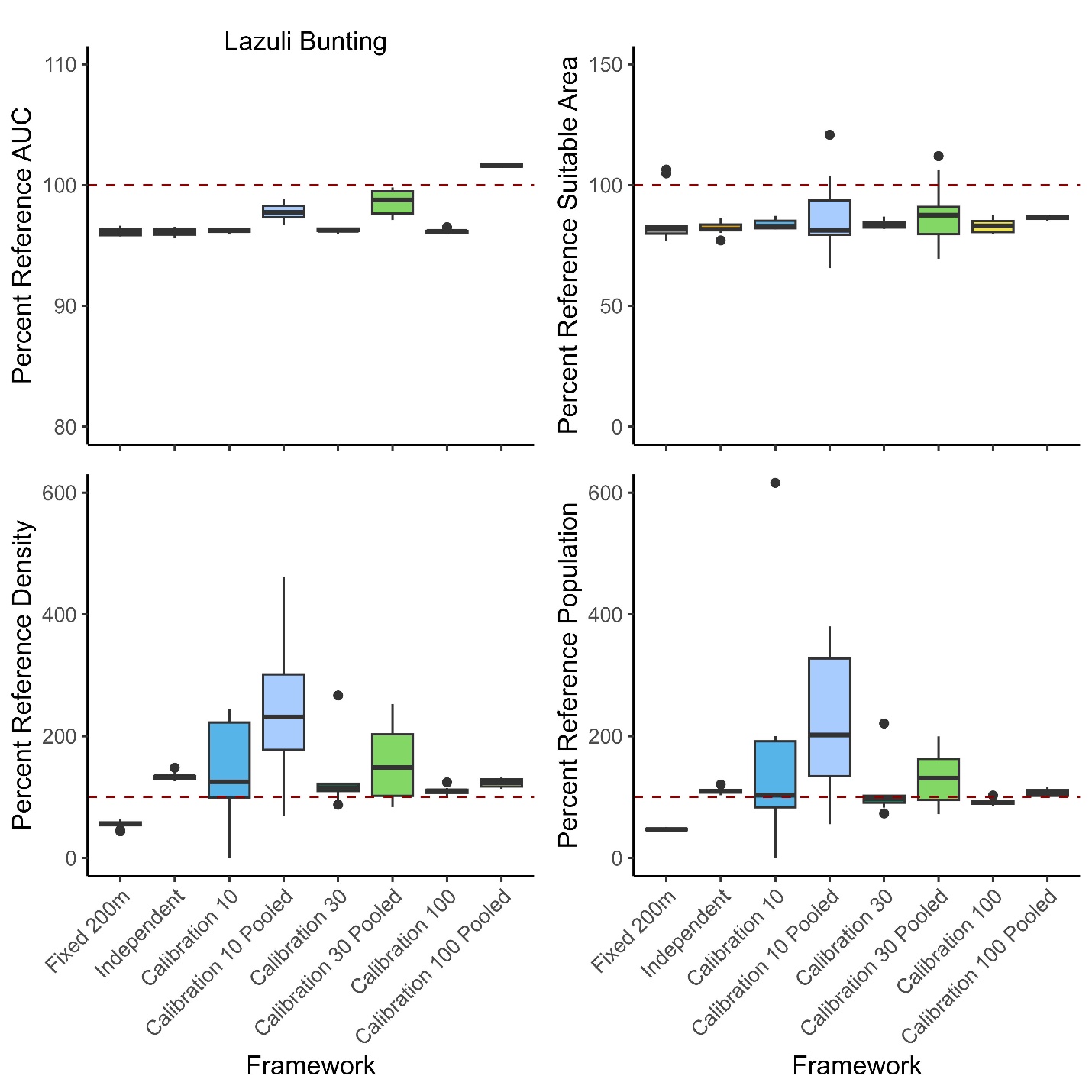


**Supplementary Figure S2.** Continued.


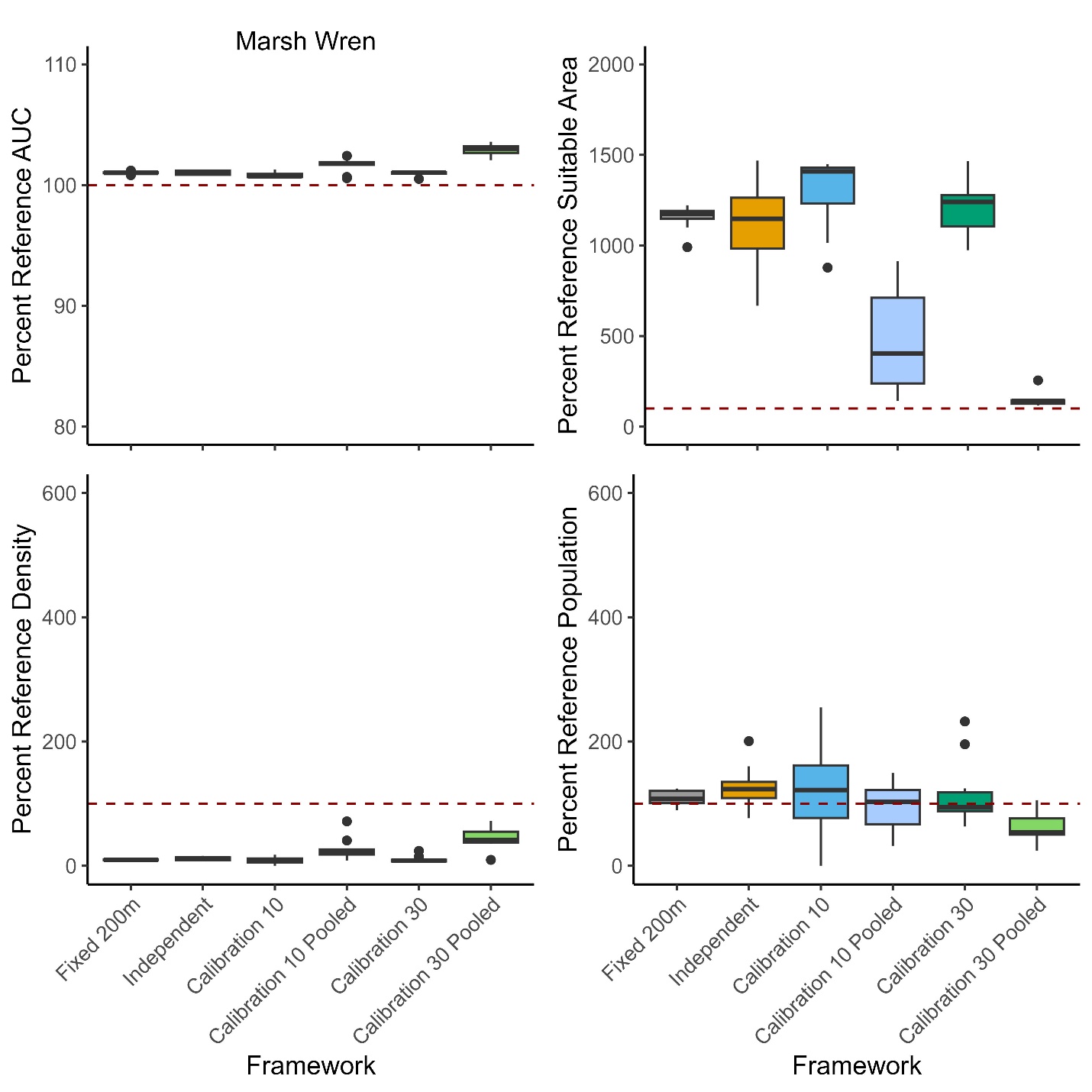


**Supplementary Figure S2.** Continued.


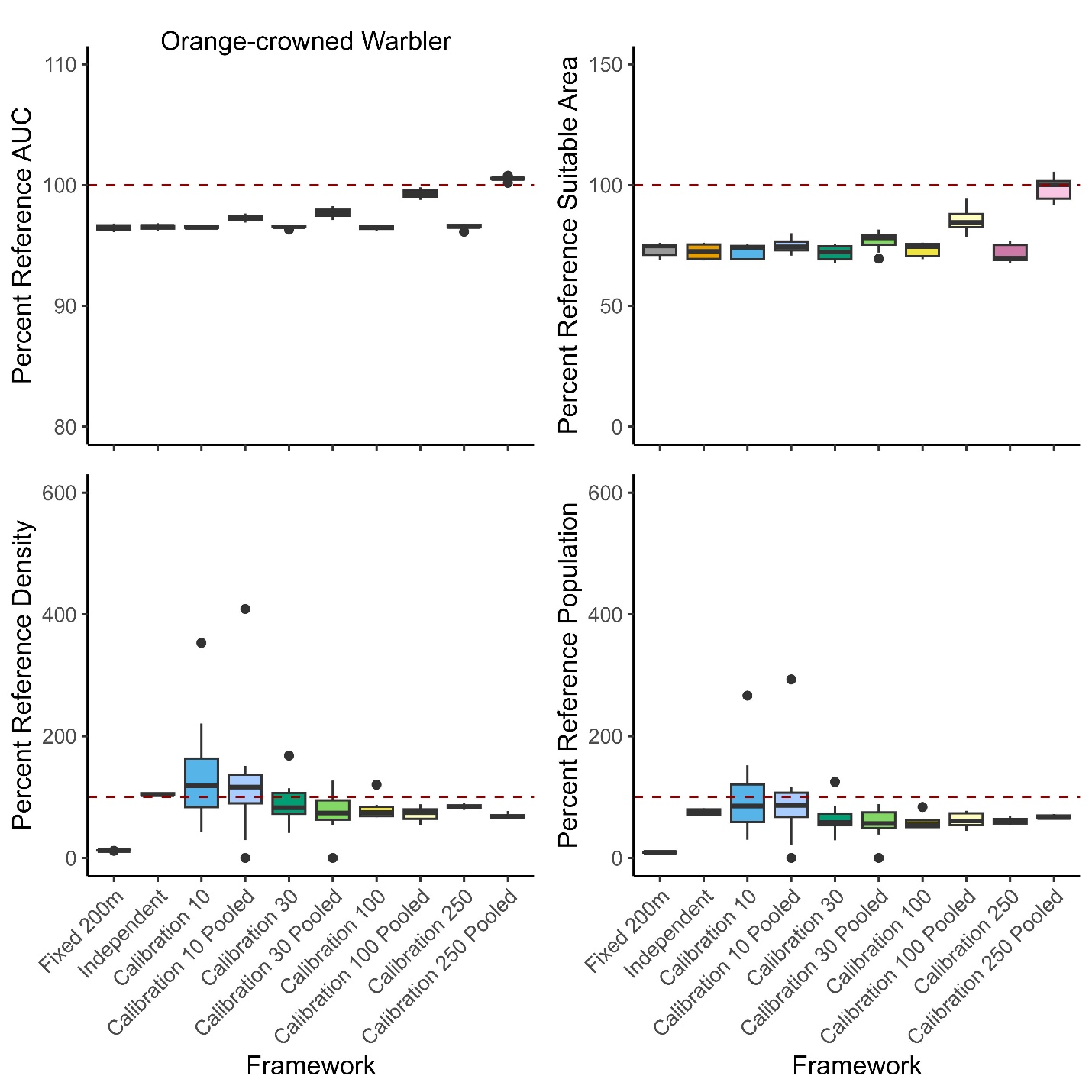


**Supplementary Figure S2.** Continued.


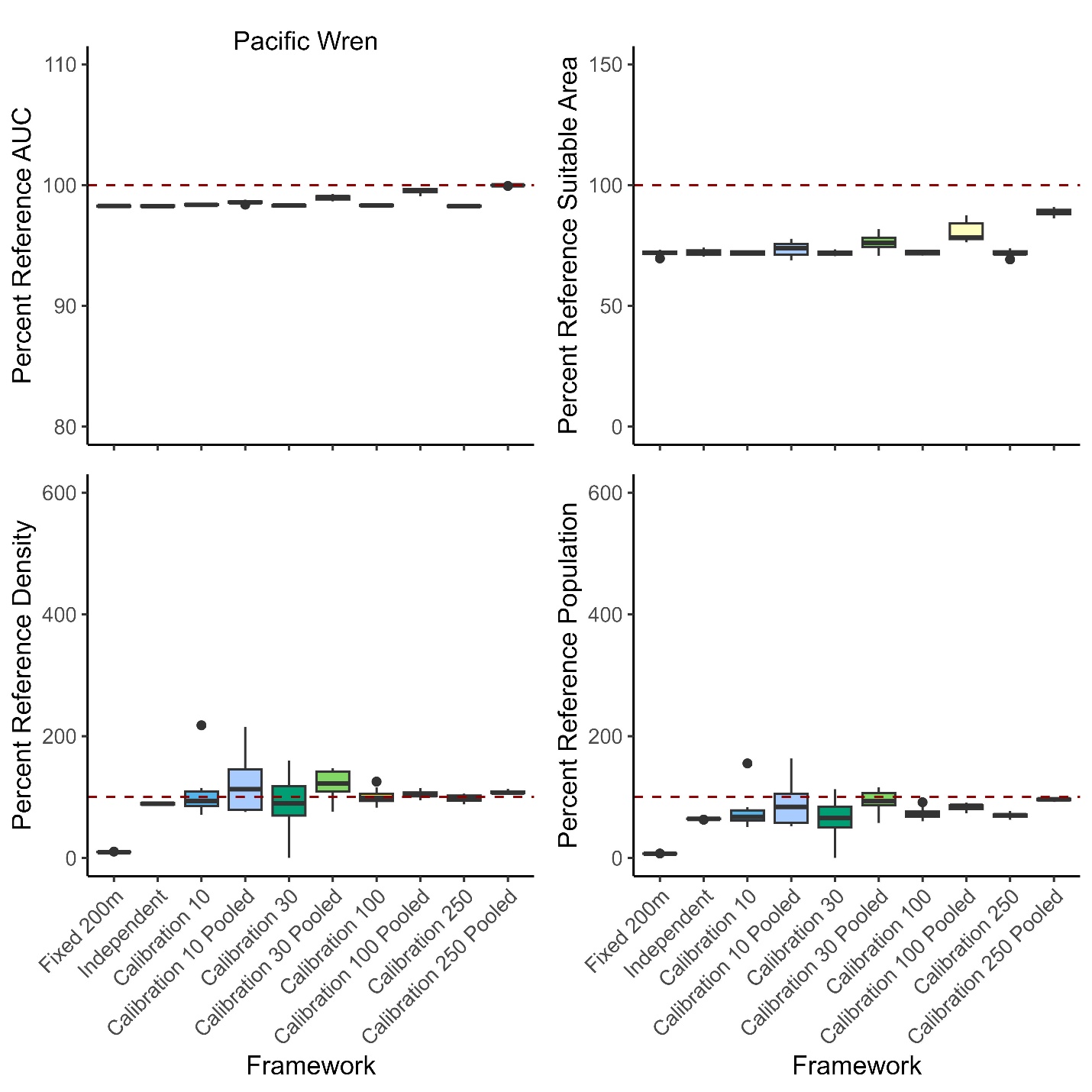


**Supplementary Figure S2.** Continued.


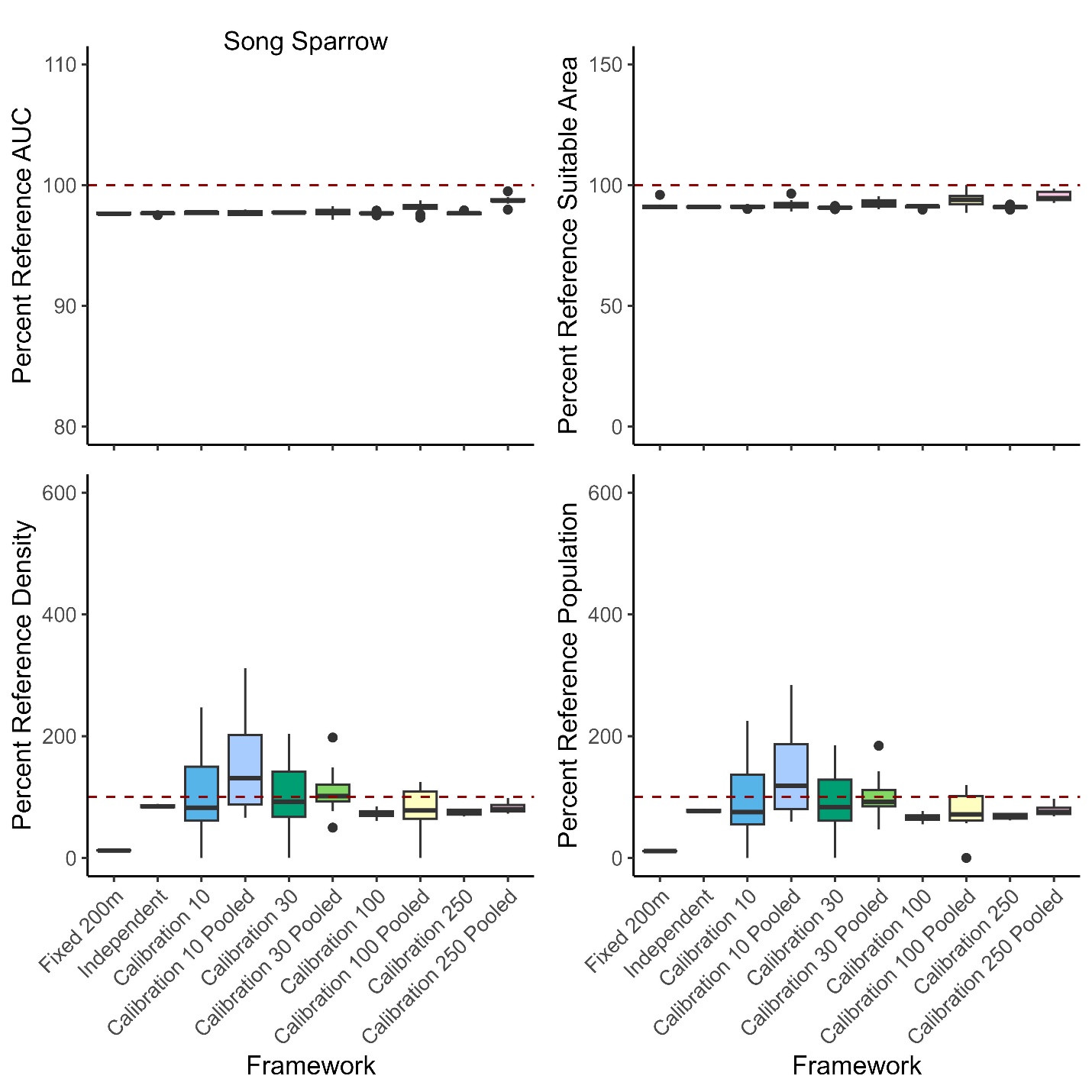


**Supplementary Figure S2.** Continued.


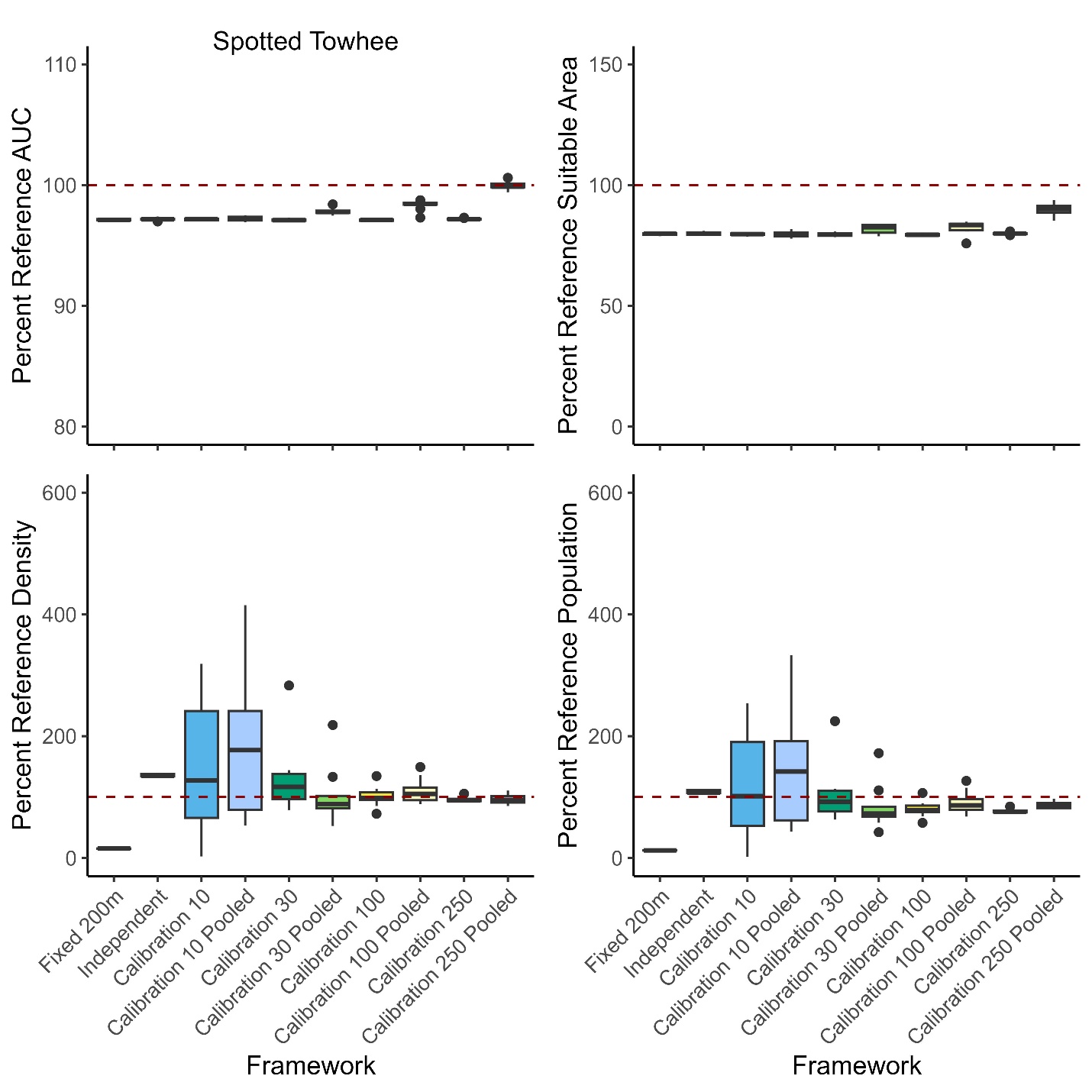


**Supplementary Figure S2.** Continued.


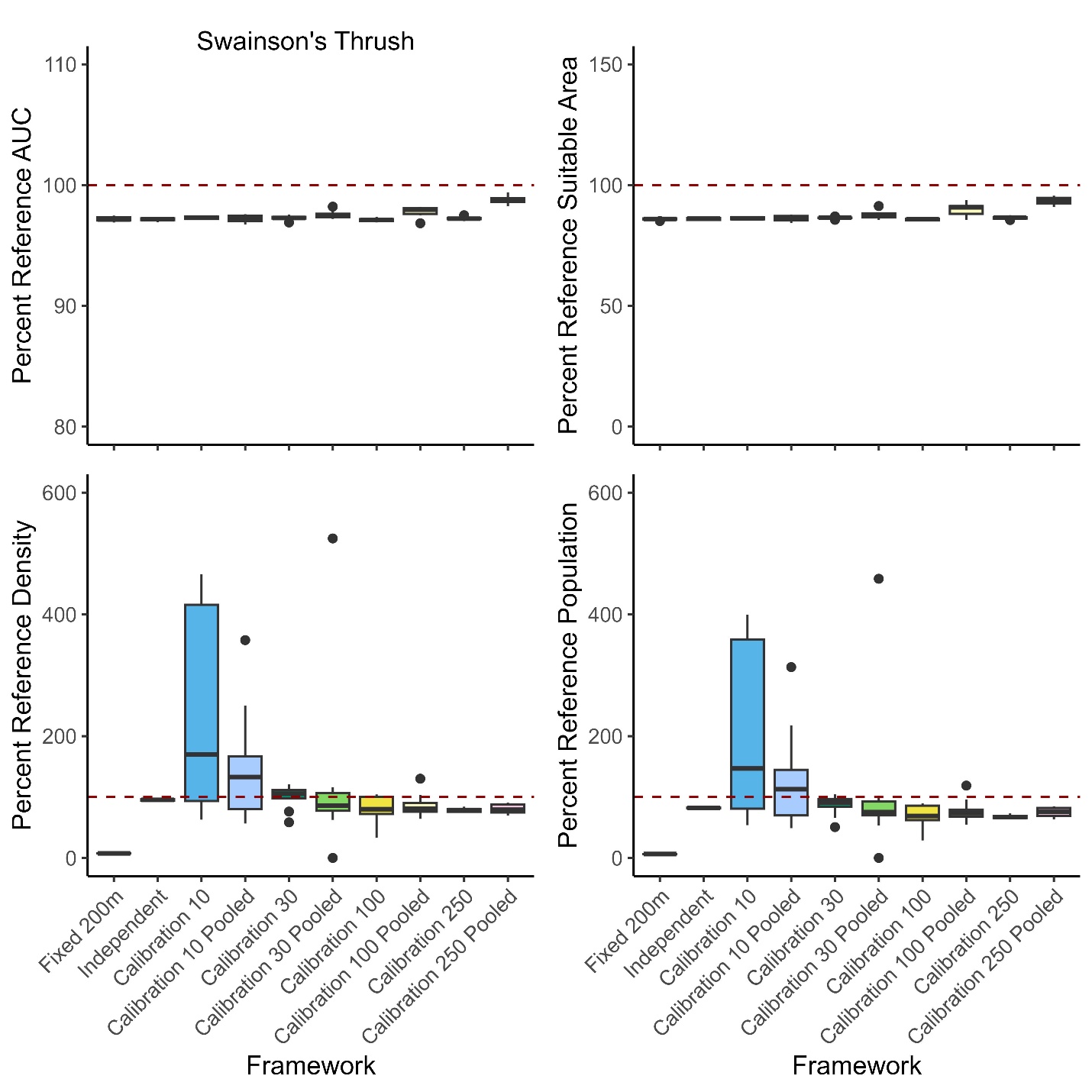


**Supplementary Figure S2.** Continued.


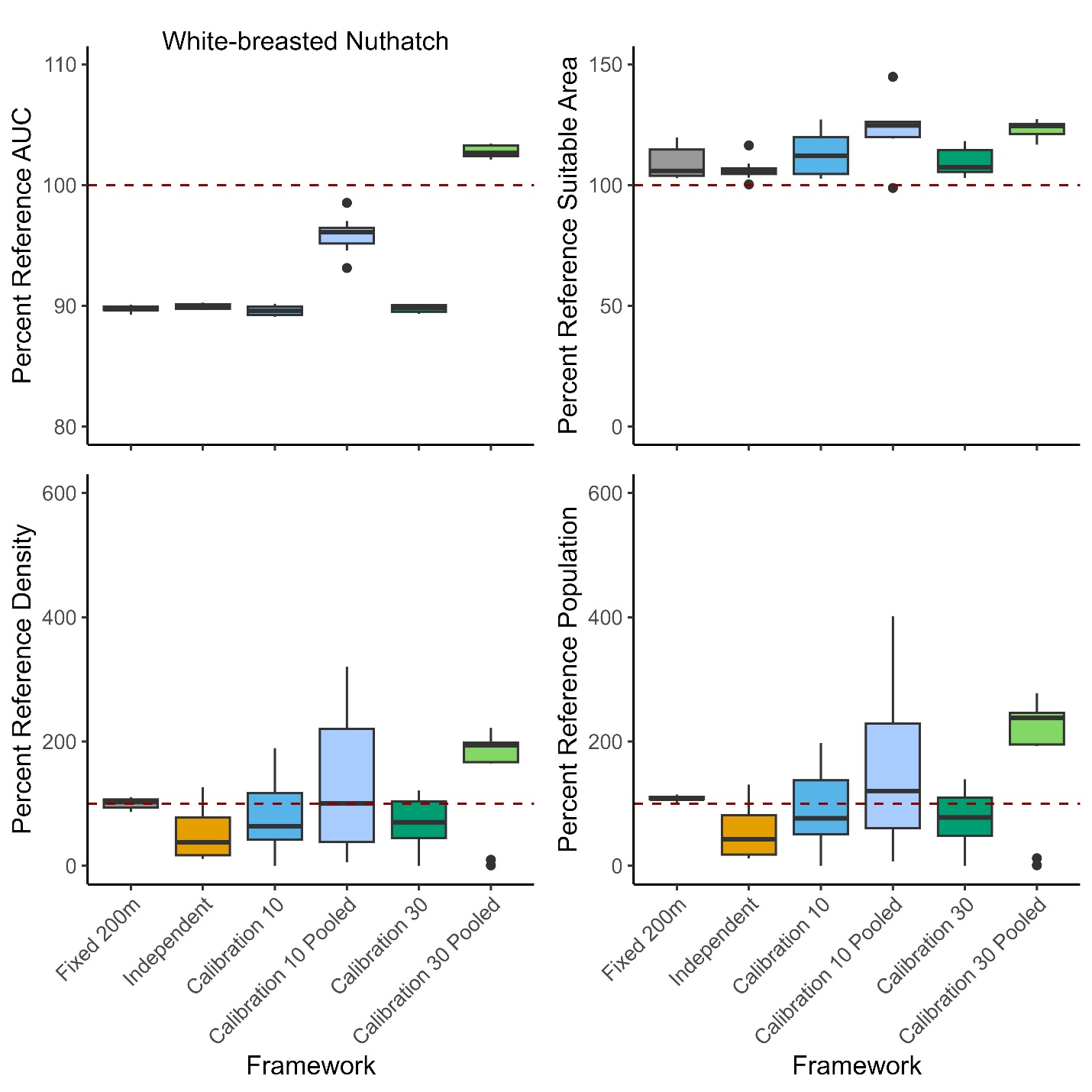


**Supplementary Figure S2.** Continued.


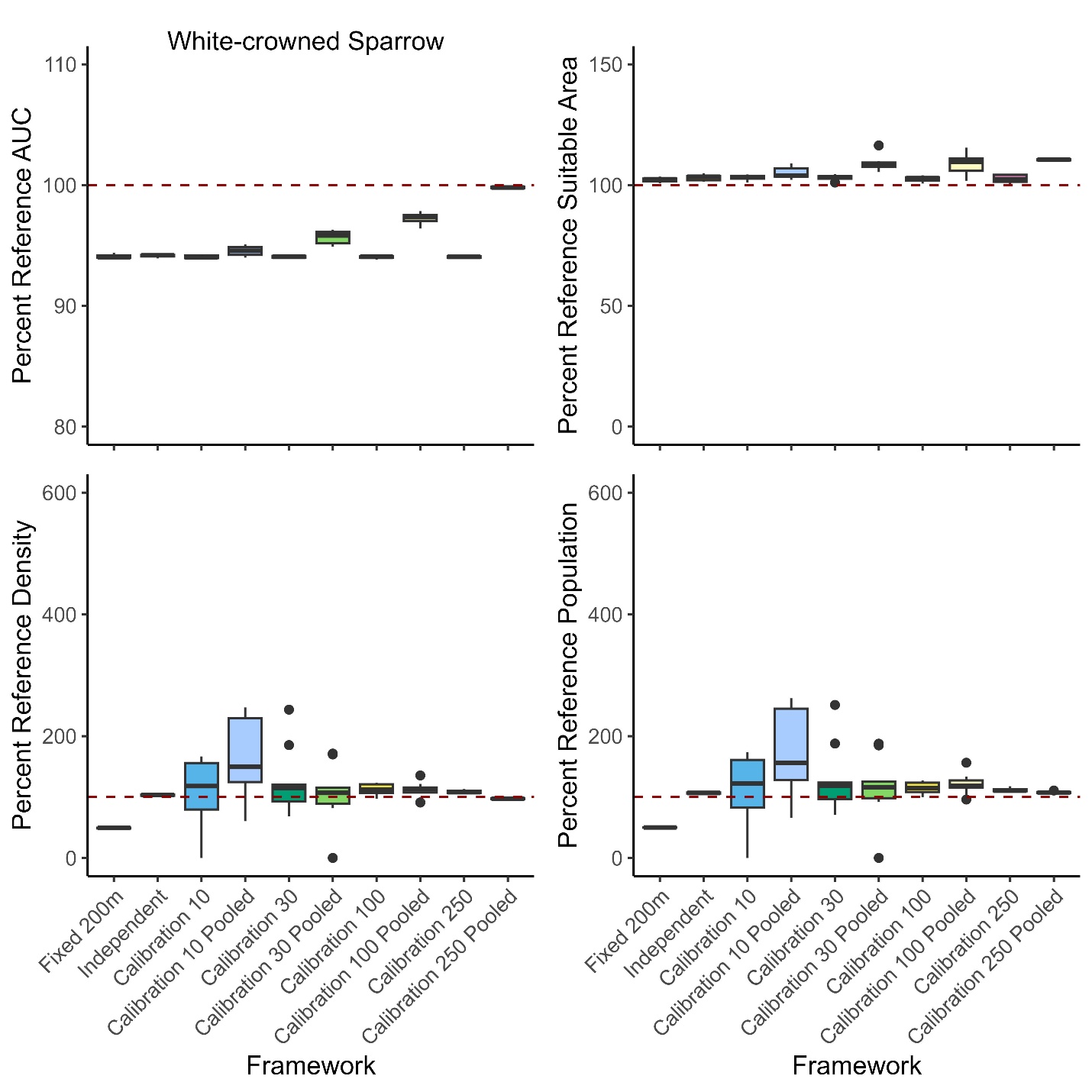


**Supplementary Figure S2.** Continued.


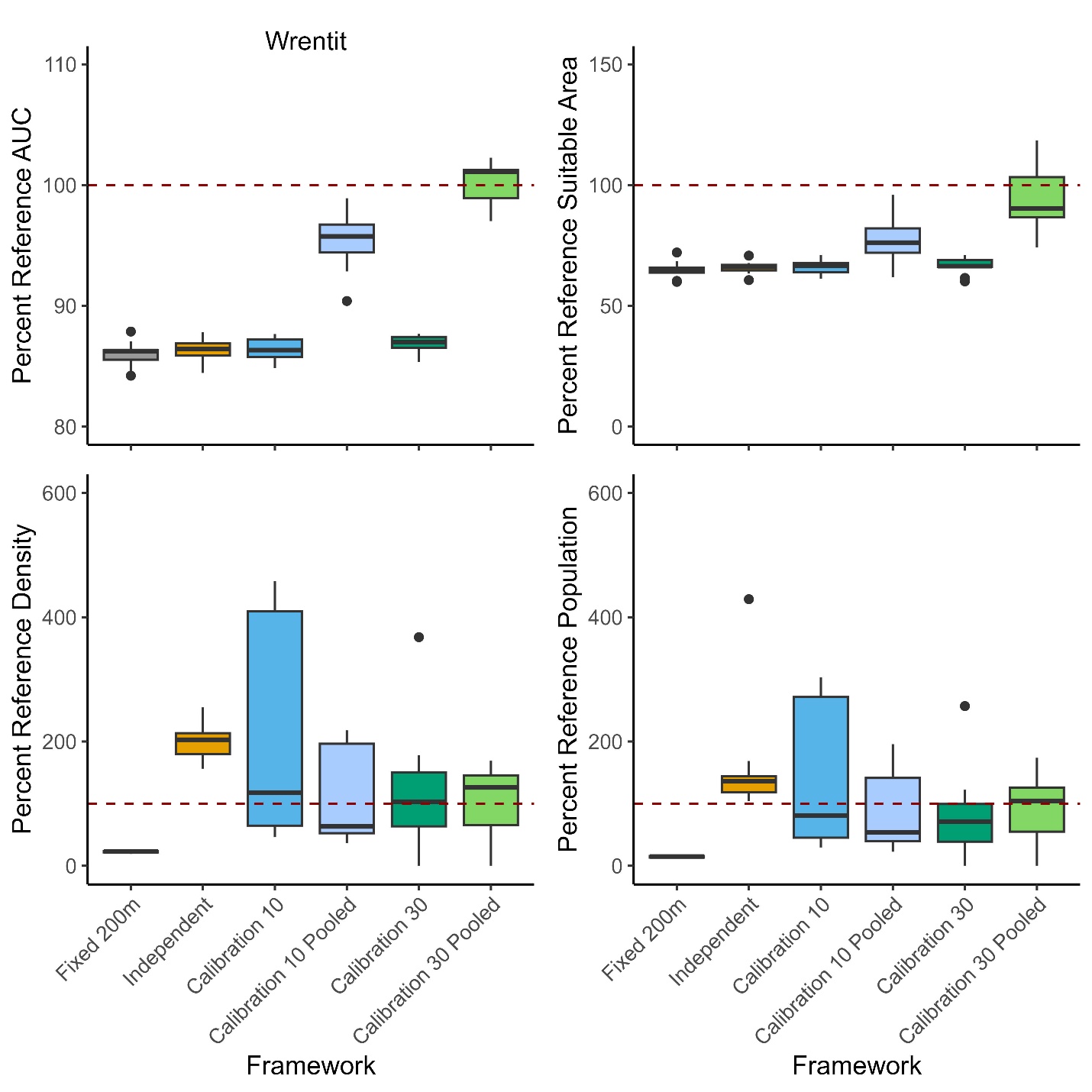


**Supplementary Figure S2.** Continued.
